# Comprehensive genomic profiling of *ESR1*,* PIK3CA*,* AKT1*, and *PTEN* in HR(+)HER2(−) metastatic breast cancer: prevalence along treatment course and predictive value for endocrine therapy resistance in real-world practice

**DOI:** 10.1007/s10549-024-07376-w

**Published:** 2024-06-14

**Authors:** Manali A. Bhave, Julia C. F. Quintanilha, Hanna Tukachinsky, Gerald Li, Takara Scott, Jeffrey S. Ross, Lincoln Pasquina, Richard S. P. Huang, Heather McArthur, Mia A. Levy, Ryon P. Graf, Kevin Kalinsky

**Affiliations:** 1 Winship Cancer Institute, Emory University, 1365 Clifton Rd NE, Building B, Suite 4000, Atlanta, GA 30322 USA; 2 Foundation Medicine, Inc, 400 Summer Street, Boston, MA 02210 USA; 3 Upstate Medical University, Syracuse, NY USA; 4 University of Texas Southwestern, Dallas, TX USA; 5 Rush University Medical Center, Chicago, IL USA

**Keywords:** HR(+)HER2(−), Metastatic breast cancer, Genomic sequencing, ESR1, AKT inhibitor

## Abstract

**Background:**

The treatment landscape for HR(+)HER2(−) metastatic breast cancer (MBC) is evolving for patients with *ESR1* mutations (mut) and PI3K/AKT pathway genomic alterations (GA). We sought to inform clinical utility for comprehensive genomic profiling (CGP) using tissue (TBx) and liquid biopsies (LBx) in HR(+)HER2(−) MBC.

**Methods:**

Records from a de-identified breast cancer clinicogenomic database for patients who underwent TBx/LBx testing at Foundation Medicine during routine clinical care at ~ 280 US cancer clinics between 01/2011 and 09/2023 were assessed. GA prevalence [*ESR1*mut, *PIK3CA*mut, *AKT1*mut, *PTEN*mut, and *PTEN* homozygous copy loss (*PTEN*loss)] were calculated in TBx and LBx [stratified by ctDNA tumor fraction (TF)] during the first three lines of therapy. Real-world progression-free survival (rwPFS) and overall survival (rwOS) were compared between groups by Cox models adjusted for prognostic factors.

**Results:**

~ 60% of cases harbored 1 + GA in 1st-line TBx (1266/2154) or LBx TF ≥ 1% (80/126) and 26.5% (43/162) in LBx TF < 1%. *ESR1*mut was found in 8.1% TBx, 17.5% LBx TF ≥ 1%, and 4.9% LBx TF < 1% in 1st line, increasing to 59% in 3rd line (LBx TF ≥ 1%). *PTEN*loss was detected at higher rates in TBx (4.3%) than LBx (1% in TF ≥ 1%). Patients receiving 1st-line aromatase inhibitor + CDK4/6 inhibitor (*n* = 573) with *ESR1*mut had less favorable rwPFS and rwOS versus *ESR1* wild-type; no differences were observed for fulvestrant + CDK4/6 inhibitor (*n* = 348).

**Conclusion:**

Our study suggests obtaining TBx for CGP at time of de novo/recurrent diagnosis, followed by LBx for detecting acquired GA in 2nd + lines. Reflex TBx should be considered when ctDNA TF < 1%.

**Supplementary Information:**

The online version contains supplementary material available at 10.1007/s10549-024-07376-w.

## Introduction

The standard of care (SOC) for HR(+)HER2(−) metastatic breast cancer (MBC) is evolving with the introduction of new biomarker-guided drugs, including novel selective estrogen receptor degraders (SERDs) for patients with *ESR1* mutations (*ESR1*mut) and PIK3CA and AKT inhibitors for patients with alterations in the PI3K/AKT pathway (*PIK3CA*, *AKT1*, and *PTEN*), as well as next-generation PI3K inhibitors. Endocrine therapy (ET) with an aromatase inhibitor (AI) is typically the first option for patients with de novo and ET-naïve MBC or for MBC tumors that have recurred at least 12 months from their adjuvant ET. A SERD is usually the first option for MBC patients with early recurrence on adjuvant AI. More recently, capivasertib, an AKT inhibitor, was approved and is now recommended by the National Comprehensive Cancer Network (NCCN) guidelines to be added to ET for patients with PI3K/AKT pathway-driven tumors, including cases with activating mutations in *AKT1* (*AKT1*mut), *PIK3CA* (*PIK3CA*mut), or *PTEN* alterations, whose disease has progressed on one or more ET-based regimens in the metastatic setting or who have experienced recurrence during or within 12 months of completing adjuvant therapy [1].

Although most HR(+)HER2(−) MBC benefit from 1st-line ET, second-line ET monotherapy has shown limited efficacy likely due to acquired resistance mechanisms [2]. *ESR1*mut has been identified as the main acquired resistance mechanism to ET [3, 4] and have been reported in approximately 20–40% of AI-treated patients with MBC varying by sites of metastatic disease [5]. *ESR1* is a transcription factor that codes for the estrogen receptor (ER) alpha protein, and *ESR1*mut may result in constitutive activation of the ER pathway unaffected by AI depletion of estrogen [2]. Guidelines from both the American Society of Clinical Oncology (ASCO) and the NCCN now recommend *ESR1*mut testing in either tissue or liquid samples at recurrence or progression on ET [1, 6] based on the EMERALD trial results which showed improved clinical outcomes with elacestrant compared to SOC endocrine therapy for patients with *ESR1*mut detected by ctDNA [7].

Several completed and ongoing clinical trials attempt to investigate the role of acquired *ESR1*mut in patients with HR(+)HER2(−) MBC receiving ET treatment and its implications for optimizing first and subsequent lines of therapy. In the PADA-1 trial, patients receiving AI + CDK 4/6 inhibitor (CDK4/6i) were screened every two months for *ESR1*mut [2]. Patients whose tumors had an *ESR1*mut detected and subsequently switched to another ET backbone before clinical radiologic disease progression had better progression-free survival (PFS) than those who only shifted treatment upon clinical progression [2].

Previous studies have shown that *ESR1*mut prevalence is only 1.5–7% in recurrent BC after prior adjuvant or neoadjuvant AI and less than 1% in ET-naïve MBC [5]. The clinical utility of *ESR1*mut detection before 1st-line MBC treatment is not well defined. *ESR1*mut prevalence is known to depend on prior duration of AI therapy, but additional data regarding the prevalence of *ESR1*mut in real-world patient populations are needed. Moreover, the emergence of novel targeted therapies, such as capivasertib for PI3K/AKT pathway-altered tumors, raises questions about the co-prevalence of alterations in *ESR1* and the PI3K/AKT pathway throughout the treatment course.

In an effort to aid optimal considerations for somatic genomic testing in HR(+)HER2(−) MBC, we sought to characterize the prevalence of *ESR1*mut and alterations in the PI3K/AKT pathway [*PIK3CA*mut, *AKT1*mut, *PTEN*mut, and *PTEN* homozygous copy loss (*PTEN*loss)] at the start of successive lines of therapy in a geographically and socioeconomically diverse real-world patient population and evaluate clinical outcomes of ET by *ESR1mut* status in 1st-line therapy in real-world practice. In addition, we report on detailed genomic alterations (GAs) in *ESR1*, *PIK3CA*, *AKT1*, and *PTEN* detected in both tissue and liquid biopsies (TBx, LBx) from MBC patients with HR(+)HER2(−).

## Methods

### Study population

This study included patients with HR(+)HER2(−) MBC who underwent genomic testing using tissue or liquid comprehensive genomic profiling (CGP) assays at Foundation Medicine during routine care. Clinical data were obtained from the nationwide (US-based) de-identified Flatiron Health and Foundation Medicine real-world clinicogenomic breast database (FH-FMI CGDB) between January 2011 and September 2023. Retrospective de-identified longitudinal clinical data were derived from electronic health records (EHR) from approximately 280 US cancer clinics (~ 800 sites of care) and comprise patient-level structured and unstructured data, curated via technology-enabled abstraction. Clinical data include demographics, clinical and laboratory features, time of therapy exposure, and survival. These were linked to genomic data derived from Foundation Medicine testing by de-identified, deterministic matching [8].

### Comprehensive genomic profiling

Hybrid capture-based NGS assays (FoundationOne®, FoundationOne®CDx, or FoundationOne®Liquid CDx) were performed on patient tumor specimens in Clinical Laboratory Improvement Amendments (CLIA)-certified, College of American Pathologists (CAP)-accredited laboratory (Foundation Medicine, Inc.). The level of ctDNA shed in the FoundationOne®Liquid CDx assay for each specimen was quantified by calculating the ctDNA tumor fraction (TF). ctDNA TF was quantified by combining multiple methods described in the Supplement 1.

### Outcomes

Real-world (rw)PFS and overall survival (rwOS) were the primary endpoints, and time to treatment discontinuation (rwTTD) was the secondary endpoint. Details of the outcome calculations are in Supplement 1.

### Data analysis

The full spectrum of predicted pathogenic *ESR1*mut, *PIK3CA*mut, *AKT1*mut, and *PTEN* GAs (encompassing mutations and homozygous copy losses) detected in TBx and LBx of patients with HR(+)HER2(−) MBC in CGDB was assessed. *ESR1*mut, *PIK3CA*mut, *AKT1*mut, *PTEN*mut, and *PTEN*loss prevalence was calculated in TBx and LBx collected in the 1st, 2nd, and 3rd lines of metastatic therapy (up to 60 days before or after start of line of treatment, not longitudinally collected). The co-occurrence of *ESR1*mut, *PIK3CA*mut, *AKT1*mut, *PTEN*mut, and *PTEN*loss was assessed in TBx collected in 1st, 2nd, and 3rd lines of therapy.

rwPFS, rwOS, and rwTTD were compared between patients with TBx who received AI + CDK4/6i and between patients receiving fulvestrant + CDK4/6i 1st-line therapy by *ESR1*mut status [*ESR1*mut versus *ESR1* wild-type (wt)] by Cox models. Multivariable analyses adjusting for age, ECOG performance status, histology, menopausal status, adjuvant therapy, bone-only versus visceral metastasis, and number of metastatic sites were performed. Chi-squared and Wilcoxon rank sum tests were used to assess differences between groups of categorical and continuous variables, respectively. Z tests were performed for comparison of the prevalence of GAs between different lines of therapy, between TBx and LBx, and between LBx with ctDNA TF < 1% versus LBx with ctDNA TF ≥ 1%. The *p*-values reported are unadjusted for multiple comparisons. The cut-off of 1% for ctDNA TF was previously determined based on sensitivity for driver alterations found in > 2000 real-world tissue/liquid pairs across multiple tumor types across ctDNA TF cut-offs [9] as well as the prevalence of fusion alterations found in tissue and liquid samples, respectively, across a database of real-world genomic results by ctDNA TF cut-offs [9–11].

R version 4.1.3 software was used for analyses. All results are to be considered and interpreted in totality, in accordance with the Bradford Hill Criteria [12] wherever possible, with no one outcome measure standing on its own.

## Results

A total of 5,780 and 1,670 HR(+)HER2(−) MBC patients with TBx and LBx, respectively, were included in this study (Supplementary Figure S1 for detailed CONSORT chart). Out of those, 1002 (17.3%) TBx and 503 (30.1%) LBx had an *ESR1*mut detected; 2,442 (42.2%) TBx and 564 (33.8%) LBx had a *PIK3CA*mut detected; 289 (5%) TBx and 70 (4.2%) LBx had an *AKT1*mut detected; and 602 (10.4%) TBx and 41 (2.5%) LBx had a *PTEN* alteration (mut or loss) detected. The most common *PTEN* alteration detected in TBx was *PTEN*loss (4.1% of all TBx samples), while in LBx, copy loss was detected in only 4 (0.2%) of the samples. The full spectrum of presumed pathogenic GAs detected in TBx and LBx are described in Supplementary Figures S2–S6.

### Prevalence of *ESR1*mut and PI3K/AKT pathway alterations (*PIK3CA*mut, AKT1mut, *PTEN*mut, and *PTEN*loss) over treatment course

**Fig. 1:**
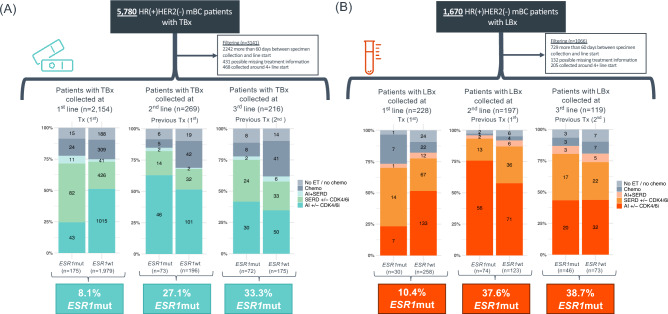
Prevalence of *ESR1*mut detected in tissue and liquid specimens of HR(+)HER2(−) mBC in the first three metastatic lines of therapy. *ESR1*mut detected in TBx (**A**) and LBx (**B**). *AI* aromatase inhibitors, *chemo* chemotherapy, *CDK4/6i* CDK 4/6 inhibitors, ET endocrine therapy, *HR* hormone receptor, *LBx* liquid biopsy, *mBC* metastatic breast cancer, *mut* mutations, *SERD* selective estrogen receptor degrader (fulvestrant), *TBx* tissue biopsy, *TF* ctDNA tumor fraction, *Tx* therapy

Evaluating specimens collected at or near the time of therapy initiation, the prevalence of *ESR1*mut in TBx collected within 60 days before or after the initiation of 1st, 2nd, and 3rd lines of therapy was 8.1% (*n* = 175/2,154), 27.1% (*n* = 73/269), and 33.3% (*n* = 72/216), respectively. Patients with *ESR1*mut detected at or near the initiation of 1st-line therapy were more likely to receive an alternative ET other than AI (TBx *p* < 0.001/LBx *p* = 0.006). Previous AI use was more common in patients with *ESR1*mut detected in 2nd and 3rd lines compared to patients with *ESR1*wt (Fig. 1A). The prevalence of *ESR1*mut in LBx collected proximal to initiation of 1st, 2nd, and 3rd lines of therapy was 10.4% (*n* = 30/288), 37.6% (*n* = 74/197), and 38.7% (*n* = 46/119), respectively (Fig. 1B).

**Fig. 2:**
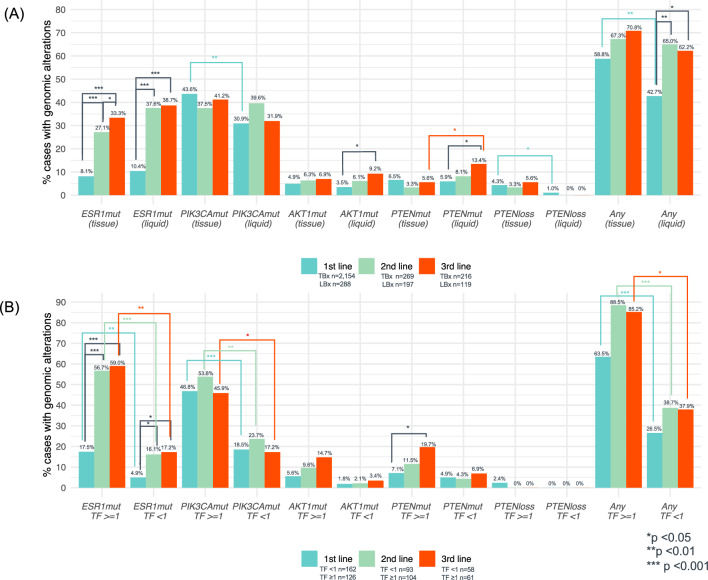
Prevalence of *ESR1*mut and PI3K/AKT pathway alterations detected in tissue and liquid specimens of HR(+)HER2(−) mBC in the first three metastatic lines of therapy. Alterations detected in TBx and LBx (**A**) and in LBx stratified by ctDNA tumor fraction (TF) (**B**). *p*-values are unadjusted. *loss* homozygous copy loss, *mut* mutation

We observed that the prevalence of an alteration in the PI3K/AKT pathway in TBx collected in 1st, 2nd, and 3rd lines of therapy was 55.1% (*n* = 1,186/2,154), 48.3% (*n* = 130/269), and 54.6% (*n* = 118/216), respectively. In LBx, the prevalence was 38.2% (*n* = 110/2,154), 49.7% (*n* = 98/269), and 46.2% (*n* = 55/216), respectively. Figure 2A shows the prevalence of each specific GA in TBx and LBx and the likelihood of detecting any of the actionable GAs (*ESR1*mut, *PIK3CA*mut, *AKT1*mut, *PTEN*mut, and *PTEN*loss) in TBx and LBx in the first three lines of therapy. We observed that 58.8% and 42.7% of patients with TBx and LBx, respectively, have at least one GA detected in 1st line, with an increase in later lines of therapy to 62–71% mainly due to the acquisition of an *ESR1*mut. We also observed that *PTEN*loss is detected at higher rates in TBx (3.3–5.6%) than LBx (0–1.0%). In an exploratory analysis to evaluate if exposure to CDK4/6 was potentially associated with some differences observed in GAs, we evaluated the prevalence of GAs in TBx samples collected prior to 2015 and in 2015 or later, and no significant differences were observed between the two groups of samples (Supplementary Figure S7). The cut-off year was chosen because the first use of a CDK4/6i was recorded in CGDB in 2015.

Given that GA detection in LBx is dependent on the tumor content of the sample, the prevalence of each GA was evaluated by the ctDNA TF status (≥ 1% versus < 1%). The prevalence of GA detected in LBx with ctDNA TF ≥ 1% was substantially higher than in LBx with ctDNA TF < 1% (Fig. 2B). Additionally, we assessed the distribution of ctDNA TF content in LBx collected from patients with bone-only metastasis versus those with visceral metastasis (with or without bone), and no difference was observed (Supplementary Figure S8).

### Co-occurrence of *ESR1*mut and PI3K/AKT pathway alterations (*PIK3CA*mut, *AKT1*mut, *PTEN*mut, and *PTEN*loss)

**Fig. 3:**
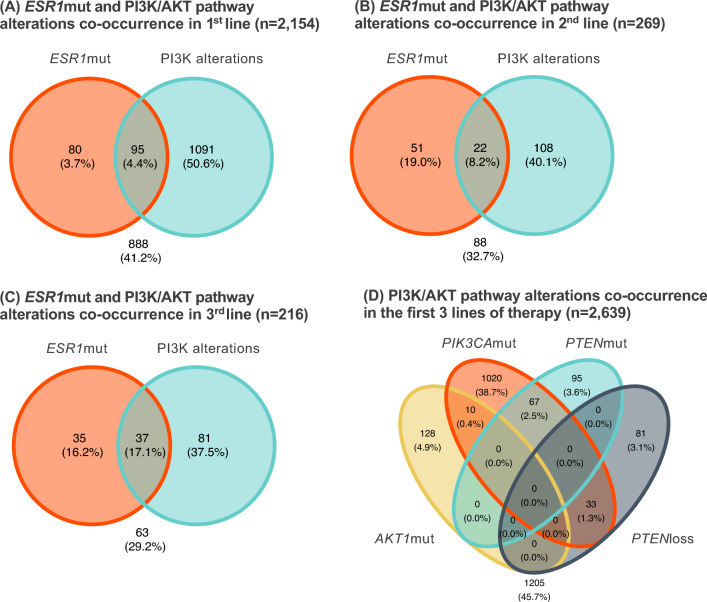
Co-occurrence of *ESR1*mut and PI3K/AKT pathway alterations detected in tissue specimens of HR(+)HER2(−) mBC in the first three metastatic lines of therapy. *loss* copy loss, *mut* mutation, PI3K/AKT alterations include *AKT1*mut, *PIK3CA*mut, *PTEN*mut, and *PTEN*loss

Among patients with TBx in 1st (*n* = 2154), 2nd (*n* = 269), and 3rd (*n* = 216) lines, both *ESR1*mut and a PI3K/AKT pathway GA were detected in 4.4% (95), 8.2% (22), and 17.1% (37), respectively (Fig. 3A).

Regarding co-alterations in the PI3K/AKT pathway, *PIK3CA*mut and *PTEN* alterations (either mut or loss) co-occurred in 4.0% (85) patients in 1st line, 1.8% (5) in 2nd line, and 4.6% (10) in 3rd line. *PTEN*loss was the only GA detected in 3.2% (68) patients in 1st line, 2.2% (6) in 2nd line, and 3.2% (7) in 3rd line. *PIK3CA*mut and *AKT1*mut co-occurred in 0.4% in 1st and 2nd lines of therapy in 0% in 3rd line. *AKT1*mut and *PTEN* alterations were mutually exclusive (Fig. 3B and Supplementary Figure S9).

### Clinical characteristic of patients with HR(+)HER2(−) MBC receiving AI + CDK4/6i or Fulvestrant + CDK4/6i in 1st-line therapy

Out of the 2,154 HR(+)HER2(−) MBC patients with TBx collected at 1st line of therapy, a total of 921 patients were included in the outcome analyses. Patients were excluded from the outcome analysis if they received therapies other than AI + CDK4/6i or fulvestrant + CDK4/6i or had CGP ordered after the start of the 2nd line of therapy (Supplementary Figure S1 for detailed CONSORT chart).

Out of the 921 patients included, 62.2% received 1st-line AI + CDK4/6i and 37.8% received Fulvestrant + CDK4/6i. Baseline *ESR1*mut was detected in 81 patients (8.8%), and of these patients, 27.2% received 1st-line AI + CDK4/6i and 72.8% received fulvestrant + CDK4/6i. The median age observed for patients with *ESR1*wt and *ESR1*mut was 63 [interquartile range (IQR) 55–70] and 65 (IQR 58–73), respectively. Most patients had an ECOG performance status of 0 (51.5%) and bone-only metastasis was observed in 32.0% of patients. Most of the cases that featured an *ESR1*mut had received adjuvant ET with AI or tamoxifen (61.7%), while only 34.4% had received adjuvant therapy with ET among those with *ESR1*wt. Finally, menopausal status and histology data were unknown in many cases, but for those cases with data available, the majority were postmenopausal and had invasive ductal histology (Table 1). See Supplementary Tables 1 and 2 for breakdown statistical comparison of baseline characteristic in patients with *ESR1*wt versus *ESR1*mut among those receiving AI + CDK4/6i and among those receiving fulvestrant + CDK4/6i in the 1st setting.

**Table 1:** Baseline patient characteristics (patients included in the outcome analyses)

	*ESR1*wt (*N* = 840)	*ESR1*mut (*N* = 81)	Total (*N* = 921)
Tx received			
AI + CDK4/6i	551 (65.6%)	22 (27.2%)	573 (62.2%)
Fulvestrant + CDK4/6i	289 (34.4%)	59 (72.8%)	348 (37.8%)
Age at Tx Start			
Median (Q1, Q3)	63.0 (55.0, 70.2)	65.0 (58.0, 73.0)	63.0 (55.0, 71.0)
Gender			
Female	829 (98.7%)	81 (100.0%)	910 (98.8%)
Male	11 (1.3%)	0 (0.0%)	11 (1.2%)
Areal socioeconomic status (quintile)			0.273
1—Lowest SES	127 (16.3%)	11 (14.1%)	138 (16.1%)
2	132 (17.0%)	11 (14.1%)	143 (16.7%)
3	158 (20.3%)	10 (12.8%)	168 (19.6%)
4	176 (22.6%)	21 (26.9%)	197 (23.0%)
5—Highest SES	185 (23.8%)	25 (32.1%)	210 (24.5%)
N-Missing	62	3	65
ECOG PS			
0	358 (51.4%)	37 (52.1%)	395 (51.5%)
1	259 (37.2%)	24 (33.8%)	283 (36.9%)
2 +	79 (11.4%)	10 (14.1%)	89 (11.6%)
N-Missing	144	10	154
Adjuvant Tx			
Adjuvant ET with AI	244 (29.0%)	49 (60.5%)	293 (31.8%)
Adjuvant ET with Tamoxifen	45 (5.4%)	1 (1.2%)	46 (5.0%)
No adjuvant ET/de novo met/unknown	551 (65.6%)	31 (38.3%)	582 (63.2%)
Menopause status*			
Postmenopausal	273 (32.5%)	44 (54.3%)	317 (34.4%)
Premenopausal	153 (18.2%)	16 (19.8%)	169 (18.3%)
Unknown	414 (49.3%)	21 (25.9%)	435 (47.2%)
Histology			
Invasive ductal carcinoma (IDC)	360 (42.9%)	46 (56.8%)	406 (44.1%)
Invasive lobular carcinoma (ILC)	93 (11.1%)	15 (18.5%)	108 (11.7%)
Other/unknown	387 (46.1%)	20 (24.7%)	407 (44.2%)
Metastasis site(s)			0.643
Bone only	270 (32.1%)	24 (29.6%)	294 (31.9%)
Visceral (CNS, liver, adrenal, other) with or without bone	570 (67.9%)	57 (70.4%)	627 (68.1%)

*AI* Aromatase inhibitors, *CDK4/6i* CDK 4/6 inhibitors, *CNS* Central nervous system, *ECOG PS* Eastern Cooperative Oncology Group performance status, ESR1mut *ESR1* mutations, ESR1wt *ESR1* wild-type, *ET* Endocrine therapy, *SES* Socioeconomic status, *Tx* Therapy

*Menopausal status only abstracted for patients diagnosed at stages I–III

### Baseline *ESR1*mut is associated with less favorable outcomes in patients receiving 1st-line AI + CDK4/6i, but not in patients receiving fulvestrant + CDK4/6i

**Fig. 4:**
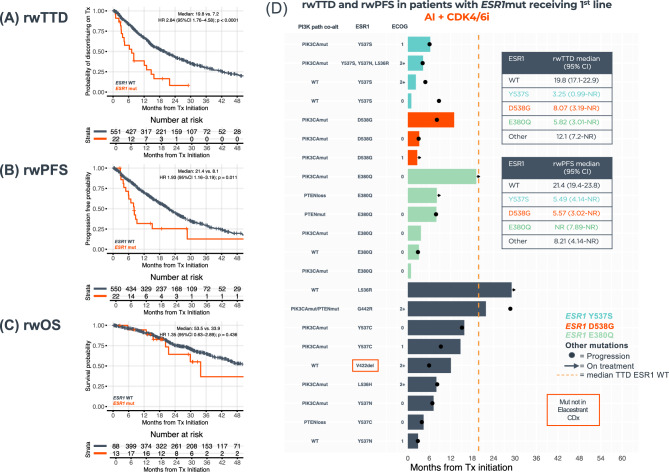
Clinical outcomes of HR(+)HER2(−) metastatic breast cancer patients receiving 1st-line AI + CDK4/6i by *ESR1*mut detected by TBx. Kaplan–Meier plots show rwTTD (**A**), rwPFS (**B**), and rwOS (**C**) for *ESR1*mut (*n* = 22) vs *ESR1*wt (*n* = 551). Swimmer plot shows rwTTD (each bar represents therapy duration on 1st line of therapy) and rwPFS (dots represent progression) for patients with *ESR1*mut ordered by specific *ERS1*mut (**D**). *AI* aromatase inhibitors, *ESR1mut ESR1* mutations, *ESR1WT* ESR1 wild-type, *HR* hazard ratio, *OS* overall survival, *PFS* progression-free survival, *rw* real-world, *TBx* tissue biopsy, *TTD* time to treatment discontinuation

**Fig. 5:**
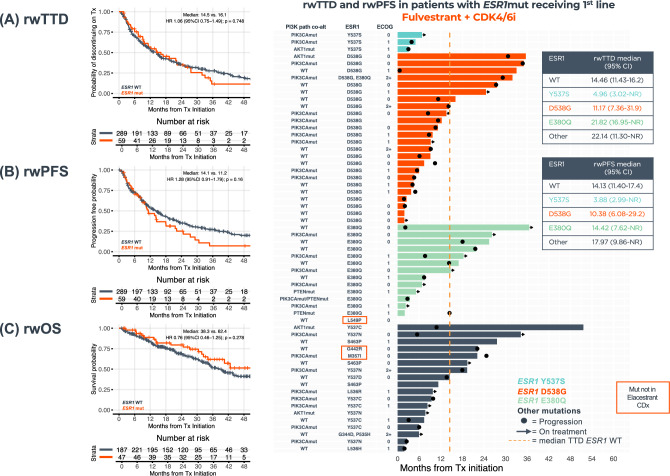
Clinical outcomes of HR(+)HER2(−) metastatic breast cancer patients receiving 1st-line Fulvestrant + CDK4/6i by *ESR1*mut detected by TBx. Kaplan–Meier plots show rwTTD (**A**), rwPFS (**B**), and rwOS (**C**) for *ESR1*mut (*n* = 59) vs *ESR1*wt (*n* = 289). Swimmer plot shows rwTTD (each bar represents therapy duration on 1st line of therapy) and rwPFS (dots represent progression) for patients with *ESR1*mut ordered by specific *ERS1*mut (**D**). *ESR1*mut *ESR1* mutations, *ESR1*WT *ESR1* wild-type, *HR* hazard ratio, *OS* overall survival, *PFS* progression-free survival, *rw* real-world, *TBx* tissue biopsy, *TTD* time to treatment discontinuation

Among patients receiving AI + CDK4/6i 1st-line therapy (*n* = 573), those with *ESR1*mut versus *ESR1*wt had less favorable rwTTD [7.2 versus 18.8 months, hazard ratio (HR) 2.84, 95% confidence interval (CI) 1.76–4.58, *p* < 0.0001], rwPFS (median 8.1 versus 21.4 months, HR 1.93, 95% CI 1.16–3.19, *p* = 0.011), and tended to have less favorable rwOS (median 33.9 versus 53.5 months, HR 1.35, 95% CI 0.63–2.89, *p* = 0.436), (Fig. 4A–C). Among patients receiving fulvestrant + CDK4/6i 1st-line therapy (*n* = 348), no difference was observed for those patients with *ESR1*mut versus *ESR1*wt (rwTTD *p* = 0.748, rwPFS *p* = 0.16, and rwOS *p* = 0.278) (Fig. 5A–C). The specific *ESR1*mut identified did not appear to impact patient outcomes with the AI + CDK4/6i regimen (Fig. 4D); however, notably shorter rwTTD and rwPFS were observed for the three patients with *ESR1* Y537S receiving fulvestrant + CDK4/6i (Fig. 5D).

We further performed multivariable analyses to control for confounding variables and found the independent association of baseline *ESR1*mut and less favorable rwTTD (HR 2.42, 95% CI 1.47–4.00, *p* < 0.001), rwPFS (HR 1.79, 95% CI 1.05–3.06, *p* = 0.033), and rwOS (HR 2.07, 95% CI 0.93–4.58, *p* = 0.074) in patients receiving 1st-line AI + CDK4/6i, but not in patients receiving Fulvestrant + CDK4/6i (Supplemental Figure S10).

## Discussion

In this study, CGP was used to characterize the prevalence of *ESR1*mut and alterations in the PI3K/AKT pathway detected by TBx and LBx at the start of successive lines of therapy in geographically and diverse patients with HR(+)HER2(−) MBC. *ESR1*mut were detected in approximately 8–10% of TBx and LBx collected at 1st-line therapy, while alterations in the PI3K/AKT pathway were detected in 38–55%. The prevalence of *ESR1*mut clearly increased across the lines of therapy, most likely due to exposure to ET, with up to 33% harboring an *ESR1*mut detected by TBx and 39% detected by LBx at time of 3rd-line therapy (59% in LBx with TF ≥ 1% and 17.2% in LBx with TF < 1%). Prior AI use was seen in the majority of patients with *ESR1*mut detected in 2nd and 3rd lines. Baseline *ESR1*mut were associated with less favorable outcomes, with worse rwTTD and rwPFS seen in patients with *ESR1*mut receiving 1st line AI + CDK4/6i versus *ESR1*wt. rwOS was also worse in patients with a baseline *ESR1*mut who received 1st-line AI + CDK4/6i, although not statistically significant.

The frequencies of *ESR1*mut found in 1st-line samples in our cohort are slightly higher than reported in the literature (4–5% in recurrent MBC and 1% in de ET-naïve MBC) [13]. This may be due to the large sample size of this study compared to prior studies with over 7,000 TBx and LBx samples included and perhaps an increase in baseline TBx collected proximal to 1st line therapy and LBx collected in different lines of therapy with newer targeted therapies for HR(+)HER2(−) MBC including SERDs and PI3K/AKT inhibitors. These findings are also clinically relevant given the less favorable outcomes including worse rwPFS for patients with baseline *ESR1*mut who were started on an AI + CDK4/6i compared to *ESR1*wt, and supports ASCO and NCCN guidelines to test for acquired *ESR1*mut in patients with recurrent disease. These data also support further investigation of the clinical utility of *ESR1* testing in patients MBC overall and serial *ESR1* testing prior to radiographic progression, given the positive results of the PADA-1 clinical trial [2]. The SERENA-6 trial will help determine whether this approach achieves clinical utility [14].

While *ESR1*mut account for approximately 50% of ET resistance cases, they alone are not the sole mechanism of ET resistance. Other mechanisms of ET resistance include alterations in PI3K-AKT-mTOR, RAS-MAPK and CDK 4/6-RB-E2F pathways, as well as *ESR1* loss, amplification and translocation [3]. Combination strategies with novel ET partnered with targeted inhibitors including CDK4/6i, PI3K/AKT inhibitors, or mTOR inhibitors have therefore shown more favorable results compared to SOC endocrine monotherapy, although further investigation is ongoing, particularly with the newer, more potent ER-targeting agents.

Our understanding of acquired resistance mechanisms and treatment of patients with MBC has improved with CGP. Specifically, CGP detects a wide spectrum of activating GAs including different mutations, insertion/deletions, copy number alterations, and rearrangements. Published literature has shown that *ESR1* Y537S-mutated tumors when compared to D538G-mutated tumors have greater resistance to fulvestrant. We did observe short rwTTD and rwPFS in the three patients with *ESR1* Y537S receiving fulvestrant + CDK4/6i and a better understanding of specific *ESR1*mut and response to newer ER-targeting agents is therefore imperative not only with the development of these agents, but also when considering sequencing in patients with continued estrogen sensitivity.

The recent approval of capivasertib for patients with *PIK3CA*mut, *AKT1*mut, *PTEN*mut, and *PTEN*loss has motivated our investigation of the prevalence of these GA in different lines of therapy in addition to *ESR1*mut. We observed a slight increase in the prevalence of *AKT1*mut and *PTEN*mut across the therapy lines, especially in LBx. LBx is usually more suitable for the detection of acquired and subclonal alterations, which would be consistent with the higher prevalence of *ESR1*mut detected in LBx compared to TBx. However, the mechanism underlying potential acquired *AKT1*mut or *PTEN*mut remains relatively unexplored, warranting further investigation to understand the observed variations in prevalence. Furthermore, we found that *PTEN*loss was detected in only three LBx collected at 1st line and it was not observed in LBx collected at 2nd and 3rd lines of therapy, while TBx exhibited a prevalence ranging from 3.3 to 5.6%. Detecting copy number alterations through CGP in LBx is inherently challenging, requiring significantly higher tumor content than what is required for short variant mutation detection [15, 16], making TBx the preferred choice for *PTEN*loss detection. It is noteworthy that *PTEN*loss is the only observed targetable alteration in 3.1% of TBx patients; consequently, patients with *PTEN*loss may be erroneously precluded from accessing targeted therapy if LBx alone is utilized without TBx evaluation with validated assays.

In this study, we also assessed the prevalence of GAs detected in LBx based on ctDNA TF content. LBx with ctDNA TF ≥ 1% showed a markedly higher prevalence of any of the GAs assessed, with 63.5% detecting at least one of the GAs, compared to only 26.5% in LBx with ctDNA TF < 1% in the 1st line of therapy. The prevalence of any GA detected in 1st-line TBx was 58.5%, implying that more than half of these alterations might be overlooked in LBx with ctDNA TF < 1%. Therefore, the evaluation of ctDNA TF is essential for accurately interpreting negative LBx results, as it informs consideration for use of a tissue specimen for CGP testing in the setting of low tumor shed of ctDNA.

While we believe this study to be one of the largest CGP studies for patients with HR(+)HER2(−) MBC, there are several limitations. Due to the retrospective nature of this study, there were fewer LBx available compared to TBx, particularly at 1st-line assessment. Additionally, no patients with serial CGP were included. We were therefore unable to draw any conclusions related to variations in acquired *ESR1*mut by specimen collected (tissue versus plasma), though there was a numerically higher prevalence of *ESR1*mut detected in LBx with ctDNA TF ≥ 1% compared to those with ctDNA TF < 1%, suggesting that tissue testing particularly for patients with ctDNA TF < 1% may be indicated. The results from our study are not automatically applicable to other CGP tests. Detection of copy number loss requires robust and specialized bioinformatics analyses than detection of other alteration classes, such as mutations and rearrangements, and existing commercially available assays do not uniformly support this capability [17–24]. A strength of our study is the use of the TBx assay that was used as the companion diagnostic test (CDx) to enable the FDA approval of capivasertib, which has gone through additional analytical and clinical validations that are not necessarily required of non-FDA-approved diagnostic tests. Finally, tumor fraction algorithms vary from platform to platform and assay to assay, and the results observed here are not automatically applicable to other tumor fraction estimations.

In summary, our cohort reveals that approximately 60% of HR(+)HER2(−) MBC cases exhibit at least one GA (*ESR1*mut and/or *PIK3CA*mut, *AKT1*mut, *PTEN*mut, and *PTEN*loss) detected by TBx or LBx with ctDNA TF ≥ 1% in the 1st-line setting, while in LBx with ctDNA TF < 1% the prevalence is 26.5%. *ESR1mut* detection in 1st-line decisions may aid treatment decisions for AI versus SERD in particular for endocrine-resistant disease. *ESR1*mut prevalence increases across treatment lines, especially in LBx, with 59% in LBx with ctDNA TF ≥ 1% in 3rd line. Ongoing studies into serial monitoring for emergent *ESR1*mut during ET treatment can potentially refine the paradigm to guide treatment strategies. Additionally, *PTEN*loss is detected at much higher rates in TBx than LBx, consistent with the known inherent limitations of ctDNA testing. Our study suggests obtaining TBx testing at time of de novo or recurrent diagnosis of HR(+)HER2(−) MBC with subsequent LBx for acquired GA in 2nd + line (especially *ESR1*mut). Reflex TBx should be considered when ctDNA TF is < 1% and no actionable GA is detected with LBx.

## Supplementary Information

Below is the link to the electronic supplementary material.

Supplementary file1 (DOCX 36 KB)

Supplementary file2 (PPTX 9102 KB)

## Data Availability

The data that support the findings of this study originated by Flatiron Health, Inc. and Foundation Medicine, Inc. Requests for data sharing by license or by permission for the specific purpose of replicating results in this manuscript can be submitted to PublicationsDataaccess@flatiron.com and cgdb-fmi@flatiron.com.
