# Correction: Comprehensive genomic profiling of *ESR1*,* PIK3CA*,* AKT1*, and *PTEN* in HR(+)HER2(−) metastatic breast cancer: prevalence along treatment course and predictive value for endocrine therapy resistance in real-world practice

**DOI:** 10.1007/s10549-024-07427-2

**Published:** 2024-07-13

**Authors:** Manali A. Bhave, Julia C. F. Quintanilha, Hanna Tukachinsky, Gerald Li, Takara Scott, Jeffrey S. Ross, Lincoln Pasquina, Richard S. P. Huang, Heather McArthur, Mia A. Levy, Ryon P. Graf, Kevin Kalinsky

**Affiliations:** 1 Winship Cancer Institute, Emory University, 1365 Clifton Rd NE, Building B, Suite 4000, Atlanta, GA 30322 USA; 2 Foundation Medicine, Inc, 400 Summer Street, Boston, MA 02210 USA; 3 Upstate Medical University, Syracuse, NY USA; 4 University of Texas Southwestern, Dallas, TX USA; 5 Rush University Medical Center, Chicago, IL USA

**Correction to: Breast Cancer Research and Treatment** 10.1007/s10549-024-07376-w

The author would like to correct the typos in Figs. 1, 3 and 4 in the online published article.

In Fig. 1, Panel A: "3.3%" should be "33.3%" and in Panel B: "10.%" should be "10.4%".

In Fig. 3, Panel D: "PI3KCAmut" should be "PIK3CAmut".

In Fig. 4 Panel C: The labels "ESR1 WT" and "ESR1 mut" should be positioned slightly higher.

The corrected Figs. 1, 3 and 4 are shown below.

**Fig. 1:**
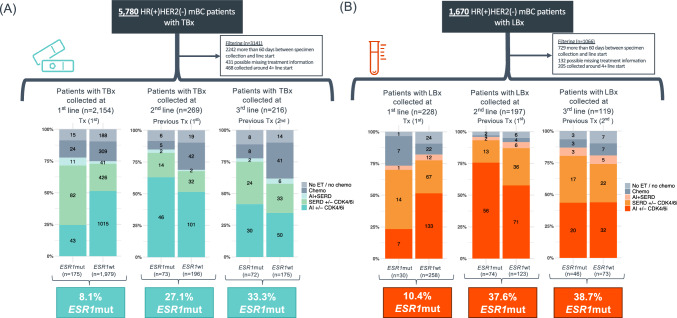
Prevalence of *ESR1*mut detected in tissue and liquid specimens of HR(+)HER2(−) mBC in the first three metastatic lines of therapy. *ESR1*mut detected in TBx (**A**) and LBx (**B**). *AI* aromatase inhibitors, *chemo* chemotherapy, *CDK4/6i* CDK 4/6 inhibitors, ET endocrine therapy, *HR* hormone receptor, *LBx* liquid biopsy, *mBC* metastatic breast cancer, *mut* mutations, *SERD* selective estrogen receptor degrader (fulvestrant), *TBx* tissue biopsy, *TF* ctDNA tumor fraction, *Tx* therapy

**Fig. 3:**
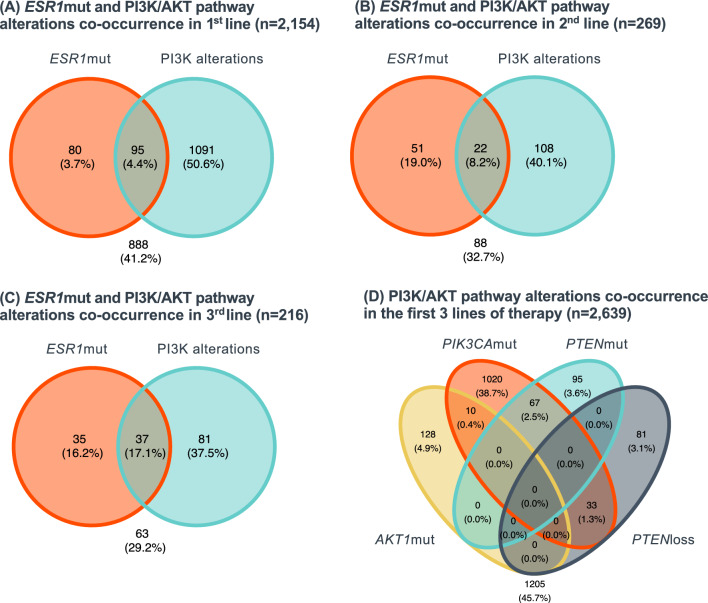
Co-occurrence of *ESR1*mut and PI3K/AKT pathway alterations detected in tissue specimens of HR(+)HER2(−) mBC in the first three metastatic lines of therapy. *loss* copy loss, *mut* mutation, PI3K/AKT alterations include *AKT1*mut, *PIK3CA*mut, *PTEN*mut, and *PTEN*loss

**Fig. 4:**
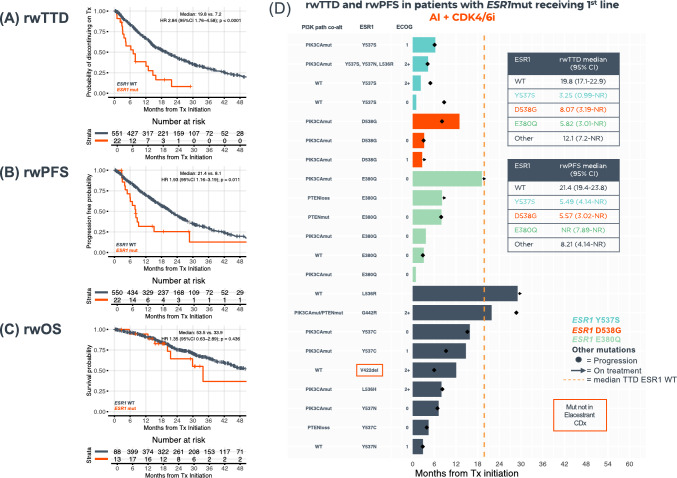
Clinical outcomes of HR(+)HER2(−) metastatic breast cancer patients receiving 1st-line AI + CDK4/6i by *ESR1*mut detected by TBx. Kaplan–Meier plots show rwTTD (**A**), rwPFS (**B**), and rwOS (**C**) for *ESR1*mut (*n* = 22) vs *ESR1*wt (*n* = 551). Swimmer plot shows rwTTD (each bar represents therapy duration on 1st line of therapy) and rwPFS (dots represent progression) for patients with *ESR1*mut ordered by specific *ERS1*mut (**D**). *AI* aromatase inhibitors, *ESR1mut ESR1* mutations, *ESR1WT* ESR1 wild-type, *HR* hazard ratio, *OS* overall survival, *PFS* progression-free survival, *rw* real-world, *TBx* tissue biopsy, *TTD* time to treatment discontinuation

The original article has been corrected.

