# Supplementary material for: Comprehensive genomic profiling of ESR1, PIK3CA, AKT1, and PTEN in HR(+)HER2(−) metastatic breast cancer: prevalence along treatment course and predictive value for endocrine therapy resistance in real-world practice
Source: Breast Cancer Res Treat. 2024 Jun 14;207(3):599–609. doi: 10.1007/s10549-024-07376-w (PMC11420341; doi:10.1007/s10549-024-07376-w)
Supplement: Supplementary file 1 — Supplementary file1 (DOCX 36 KB) [file 10549_2024_7376_MOESM1_ESM.docx]

**Supplementary Methods**

*Outcomes measurement*

rwPFS was calculated from the treatment start date until the time of disease progression or death, and patients not yet reaching progression or death were right-censored at the date of the last clinical note. rwOS was calculated from the start of treatment to death from any cause, and patients with no record of mortality were right-censored at the date of the last clinic visit or structured EHR activity. rwOS risk intervals were left truncated to the date of the CGP report to account for immortal time, as patients cannot enter the database until a CGP report is provided [1, 2]. rwTTD was calculated from the treatment start date until evidence of treatment discontinuation for any reason or death, and patients not yet reaching treatment discontinuation or death were right-censored at the date of last clinical visit, laboratory result, or medication use. The mortality information in the Flatiron Health database is a composite derived from de‐identified patient‐level data within the EHR, the public Social Security Death Index, and a commercial death dataset mining data from obituaries and funeral homes. This mortality information has been externally validated in comparison to the National Death Index[3]. In addition, the Flatiron Health and Foundation Medicine CGDB has replicated associations with survival observed in biomarker subgroup analyses of randomized controlled trials [4, 5].

*ctDNA Tumor Fraction calculation*

ctDNA TF was quantified by combining multiple methods. For samples with significant aneuploidy, the purity assessment from a robust copy-number model that accounts for both the observed coverage variation and allele frequencies of genome-wide SNP allele frequencies is used to determine the ctDNA TF estimate [6]. When significant aneuploidy is not present, the allele frequencies of short variants and rearrangements deemed very likely to be somatic are used to estimate ctDNA TF [7].

**References**

1. McGough, S.F., et al., *Penalized regression for left-truncated and right-censored survival data.* Stat Med, 2021. **40**(25): p. 5487-5500.

2. Brown, S., et al., *Implications of Selection Bias Due to Delayed Study Entry in Clinical Genomic Studies.* JAMA Oncol, 2022. **8**(2): p. 287-291.

3. Zhang, Q., et al., *Validation analysis of a composite real-world mortality endpoint for patients with cancer in the United States.* Health Serv Res, 2021. **56**(6): p. 1281-1287.

4. Quintanilha, J.C.F., et al., *Comparative Effectiveness of Immune Checkpoint Inhibitors vs Chemotherapy in Patients With Metastatic Colorectal Cancer With Measures of Microsatellite Instability, Mismatch Repair, or Tumor Mutational Burden.* JAMA Netw Open, 2023. **6**(1): p. e2252244.

5. Graf, R.P., et al., *Comparative Effectiveness of Immune Checkpoint Inhibitors vs Chemotherapy by Tumor Mutational Burden in Metastatic Castration-Resistant Prostate Cancer.* JAMA Netw Open, 2022. **5**(3): p. e225394.

6. Tukachinsky, H., et al., *Genomic Analysis of Circulating Tumor DNA in 3,334 Patients with Advanced Prostate Cancer Identifies Targetable BRCA Alterations and AR Resistance Mechanisms.* Clin Cancer Res, 2021. **27**(11): p. 3094-3105.

7. Rolfo, C.D., et al., *Utility of ctDNA tumor fraction to inform negative liquid biopsy (LBx) results and need for tissue reflex in advanced non-small cell lung cancer (aNSCLC).* Journal of Clinical Oncology, 2023. **41**(16_suppl): p. 9076-9076.
