# Supplementary material for: Comprehensive genomic profiling of ESR1, PIK3CA, AKT1, and PTEN in HR(+)HER2(−) metastatic breast cancer: prevalence along treatment course and predictive value for endocrine therapy resistance in real-world practice
Source: Breast Cancer Res Treat. 2024 Jun 14;207(3):599–609. doi: 10.1007/s10549-024-07376-w (PMC11420341; doi:10.1007/s10549-024-07376-w)
Supplement: Supplementary file 2 — Supplementary file2 (PPTX 9102 KB) [file 10549_2024_7376_MOESM2_ESM.pptx]

## Slide 1
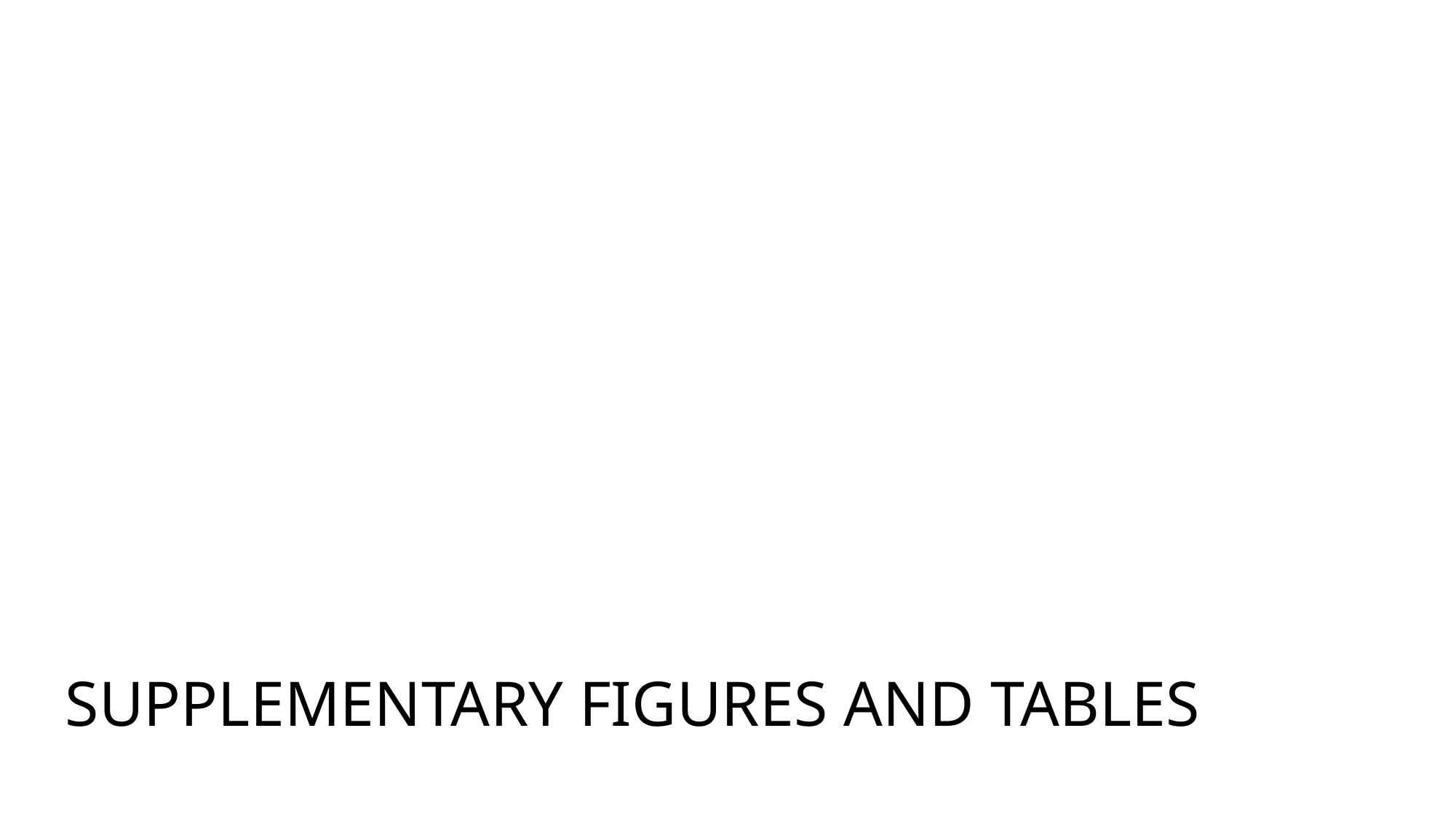

# SUPPLEMENTARY FIGURES AND TABLES

## Slide 2
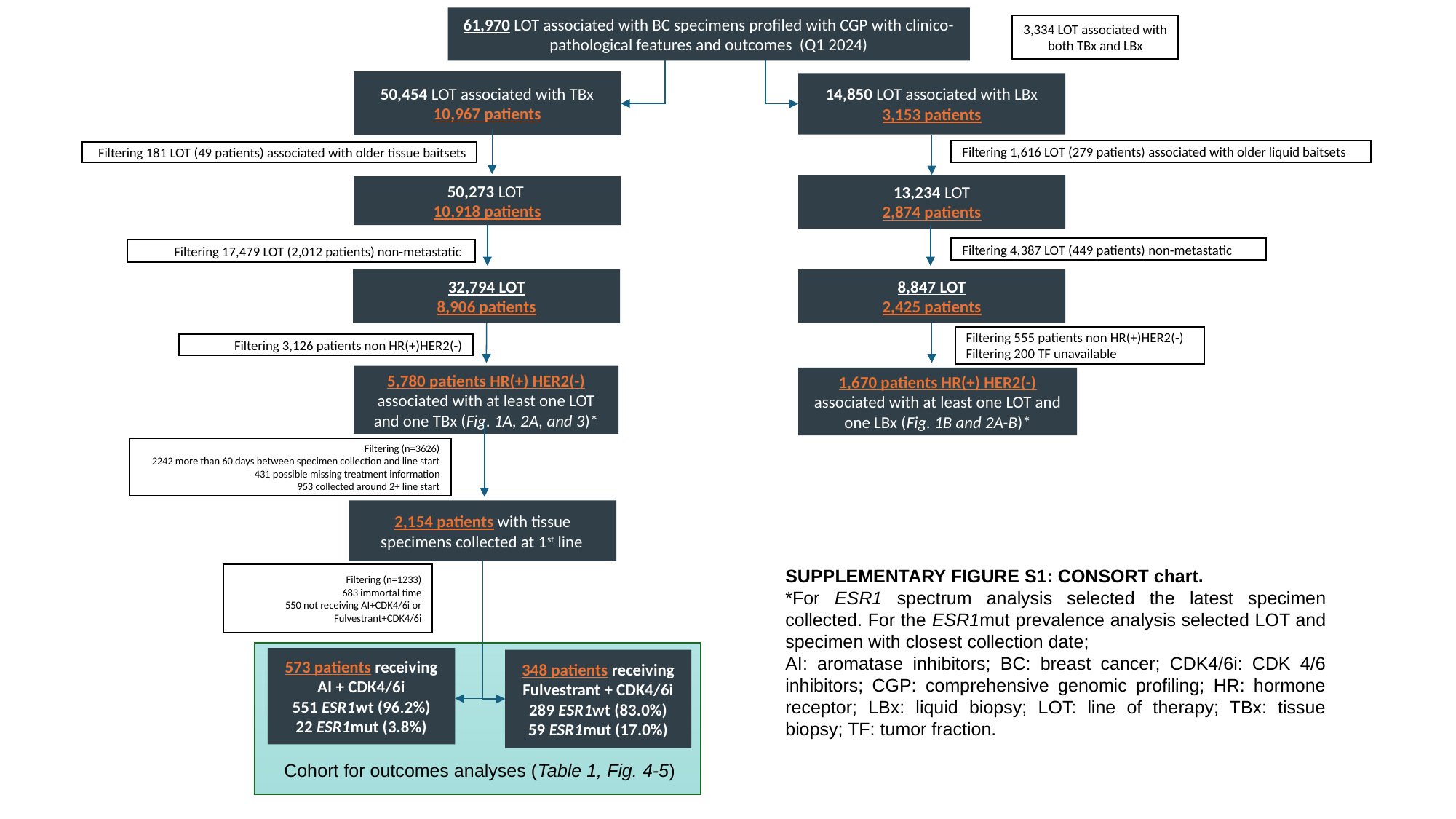

61,970 LOT associated with BC specimens profiled with CGP with clinico-pathological features and outcomes (Q1 2024)
3,334 LOT associated with both TBx and LBx
50,454 LOT associated with TBx
10,967 patients
14,850 LOT associated with LBx
3,153 patients
Filtering 1,616 LOT (279 patients) associated with older liquid baitsets
Filtering 181 LOT (49 patients) associated with older tissue baitsets
13,234 LOT
2,874 patients
50,273 LOT
10,918 patients
Filtering 4,387 LOT (449 patients) non-metastatic
Filtering 17,479 LOT (2,012 patients) non-metastatic
32,794 LOT
8,906 patients
8,847 LOT
2,425 patients
Filtering 555 patients non HR(+)HER2(-)
Filtering 200 TF unavailable
Filtering 3,126 patients non HR(+)HER2(-)
5,780 patients HR(+) HER2(-)
associated with at least one LOT and one TBx (Fig. 1A, 2A, and 3)*
1,670 patients HR(+) HER2(-)
associated with at least one LOT and one LBx (Fig. 1B and 2A-B)*
Filtering (n=3626)
2242 more than 60 days between specimen collection and line start
431 possible missing treatment information
953 collected around 2+ line start
2,154 patients with tissue specimens collected at 1st line
SUPPLEMENTARY FIGURE S1: CONSORT chart.
*For ESR1 spectrum analysis selected the latest specimen collected. For the ESR1mut prevalence analysis selected LOT and specimen with closest collection date;
AI: aromatase inhibitors; BC: breast cancer; CDK4/6i: CDK 4/6 inhibitors; CGP: comprehensive genomic profiling; HR: hormone receptor; LBx: liquid biopsy; LOT: line of therapy; TBx: tissue biopsy; TF: tumor fraction.
Filtering (n=1233)
683 immortal time
550 not receiving AI+CDK4/6i or Fulvestrant+CDK4/6i
573 patients receiving AI + CDK4/6i
551 ESR1wt (96.2%)
22 ESR1mut (3.8%)
348 patients receiving Fulvestrant + CDK4/6i
289 ESR1wt (83.0%)
59 ESR1mut (17.0%)
Cohort for outcomes analyses (Table 1, Fig. 4-5)

## Slide 3
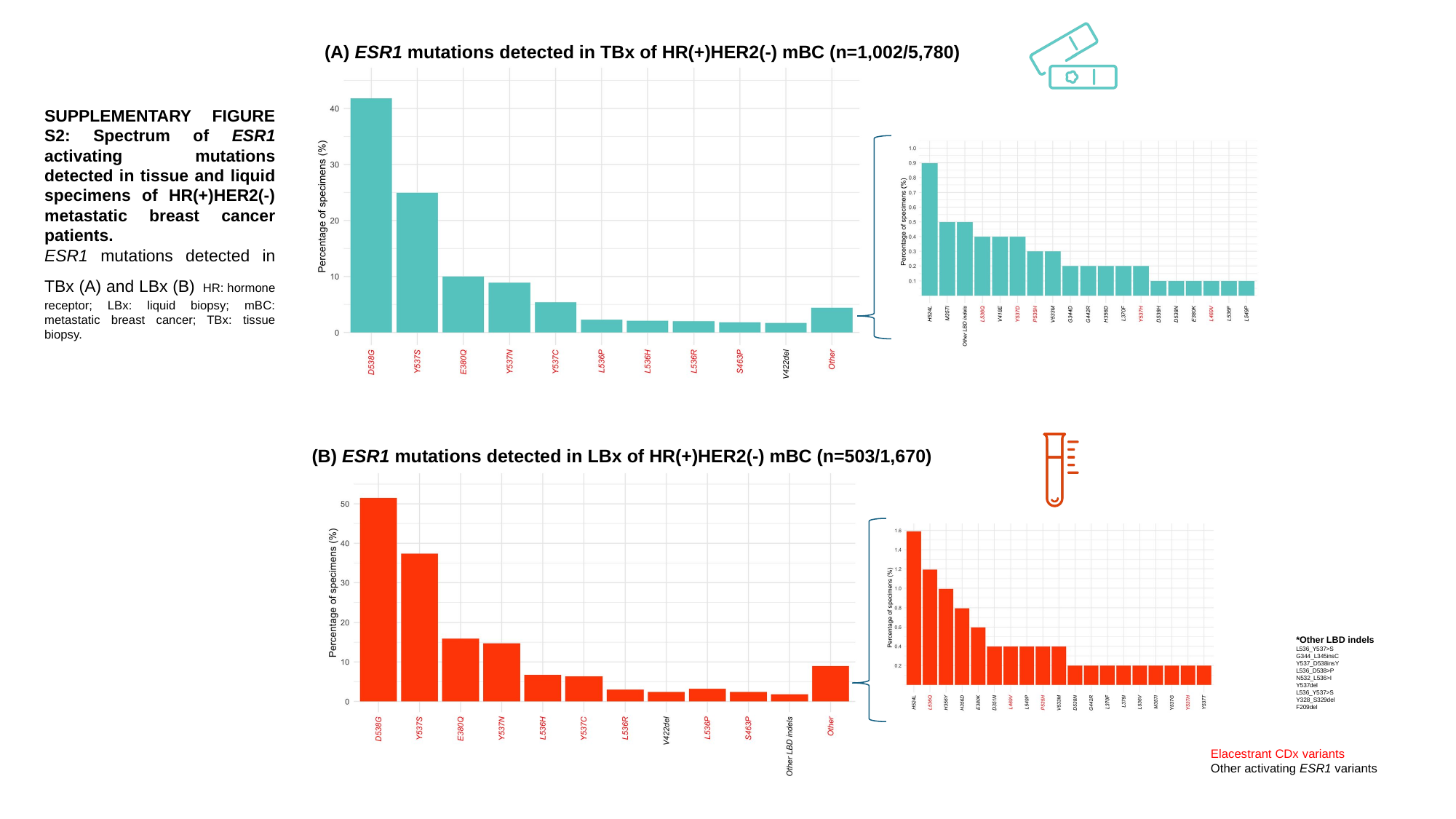

(A) ESR1 mutations detected in TBx of HR(+)HER2(-) mBC (n=1,002/5,780)
SUPPLEMENTARY FIGURE S2: Spectrum of ESR1 activating mutations detected in tissue and liquid specimens of HR(+)HER2(-) metastatic breast cancer patients.
ESR1 mutations detected in TBx (A) and LBx (B) HR: hormone receptor; LBx: liquid biopsy; mBC: metastatic breast cancer; TBx: tissue biopsy.
(B) ESR1 mutations detected in LBx of HR(+)HER2(-) mBC (n=503/1,670)
*Other LBD indels
L536_Y537>S
G344_L345insC
Y537_D538insY
L536_D538>P
N532_L536>I
Y537del
L536_Y537>S
Y328_S329del
F209del
Elacestrant CDx variants
Other activating ESR1 variants

## Slide 4
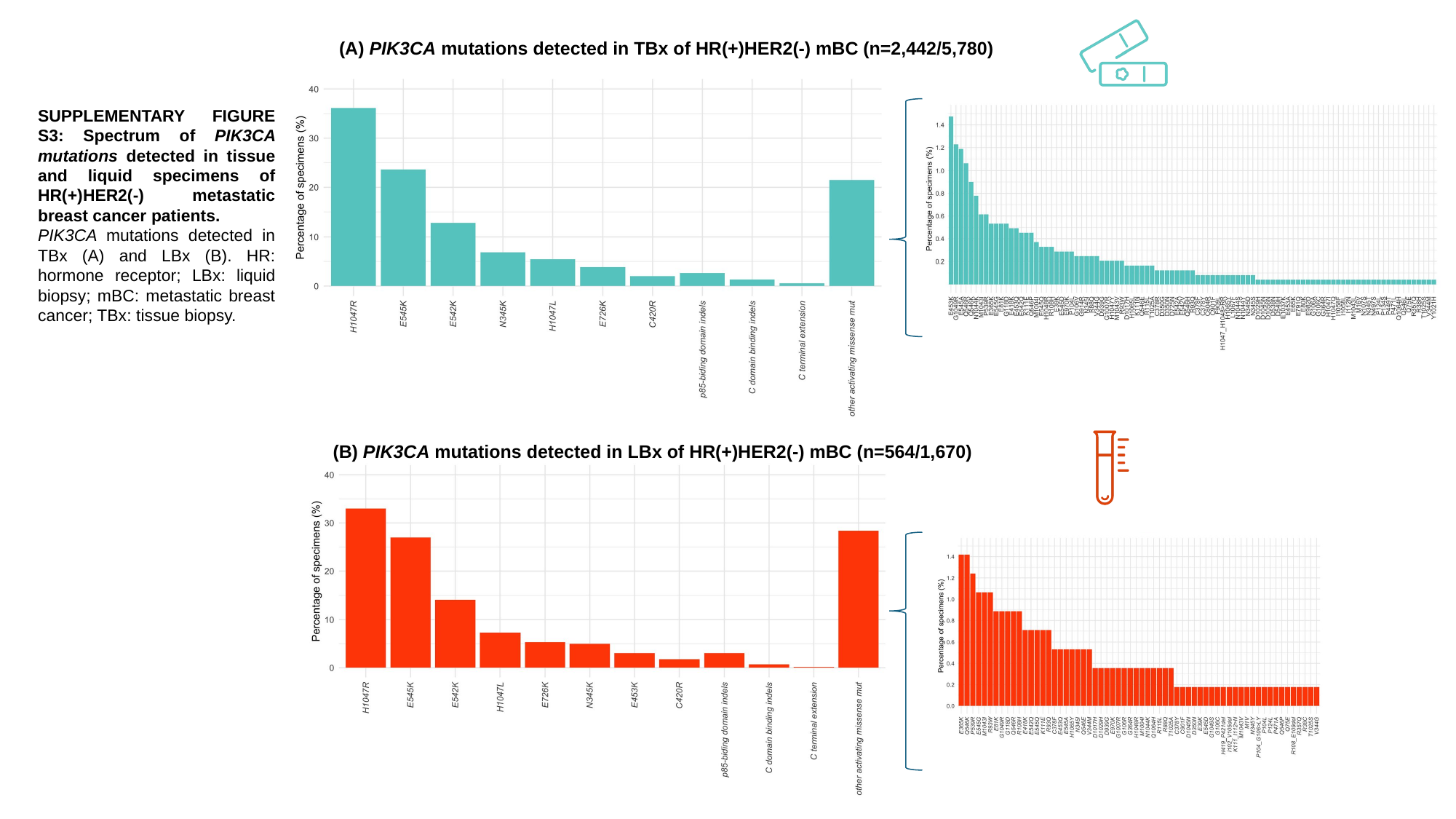

(A) PIK3CA mutations detected in TBx of HR(+)HER2(-) mBC (n=2,442/5,780)
SUPPLEMENTARY FIGURE S3: Spectrum of PIK3CA mutations detected in tissue and liquid specimens of HR(+)HER2(-) metastatic breast cancer patients.
PIK3CA mutations detected in TBx (A) and LBx (B). HR: hormone receptor; LBx: liquid biopsy; mBC: metastatic breast cancer; TBx: tissue biopsy.
(B) PIK3CA mutations detected in LBx of HR(+)HER2(-) mBC (n=564/1,670)

## Slide 5
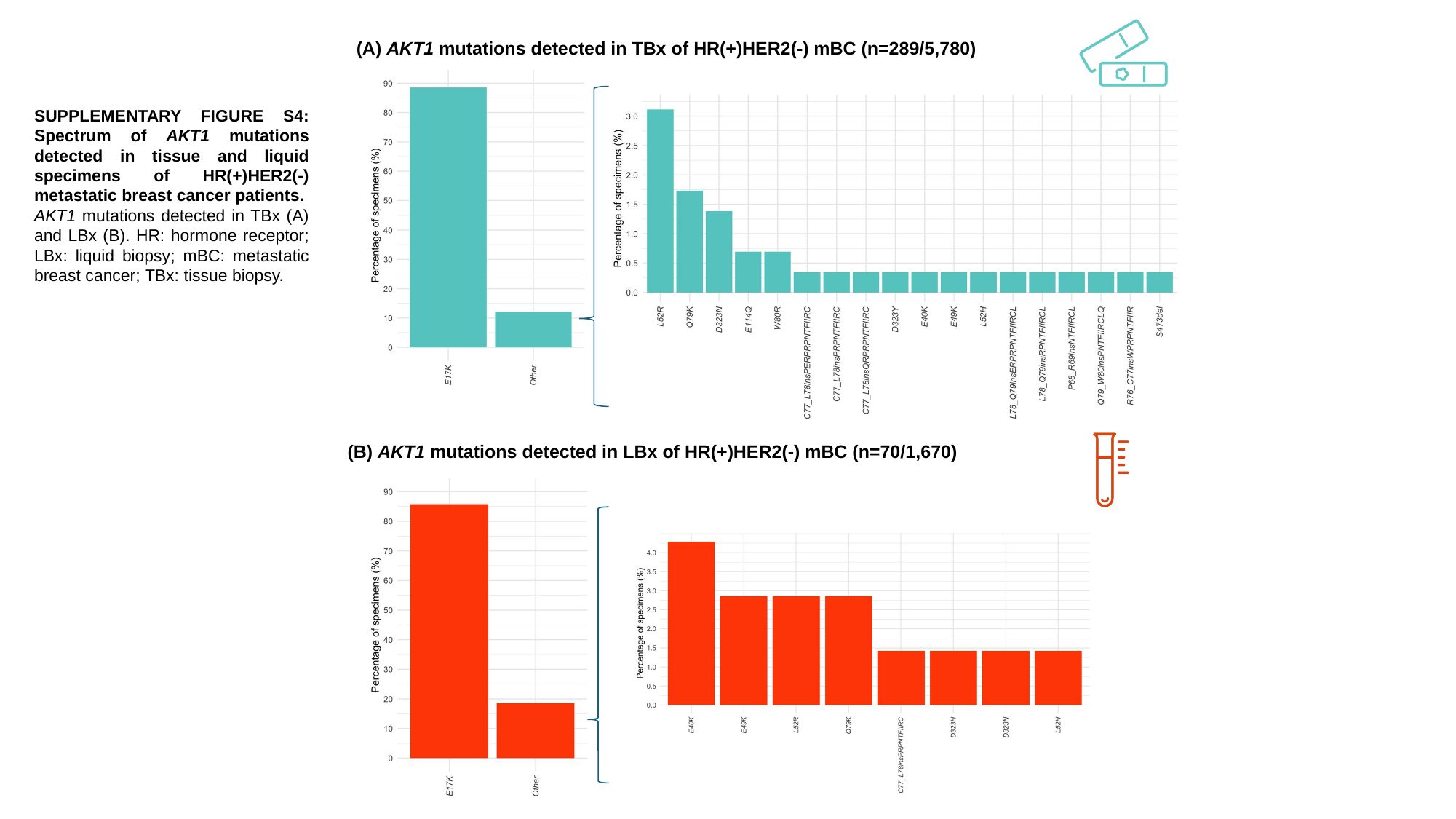

(A) AKT1 mutations detected in TBx of HR(+)HER2(-) mBC (n=289/5,780)
SUPPLEMENTARY FIGURE S4: Spectrum of AKT1 mutations detected in tissue and liquid specimens of HR(+)HER2(-) metastatic breast cancer patients.
AKT1 mutations detected in TBx (A) and LBx (B). HR: hormone receptor; LBx: liquid biopsy; mBC: metastatic breast cancer; TBx: tissue biopsy.
(B) AKT1 mutations detected in LBx of HR(+)HER2(-) mBC (n=70/1,670)

## Slide 6
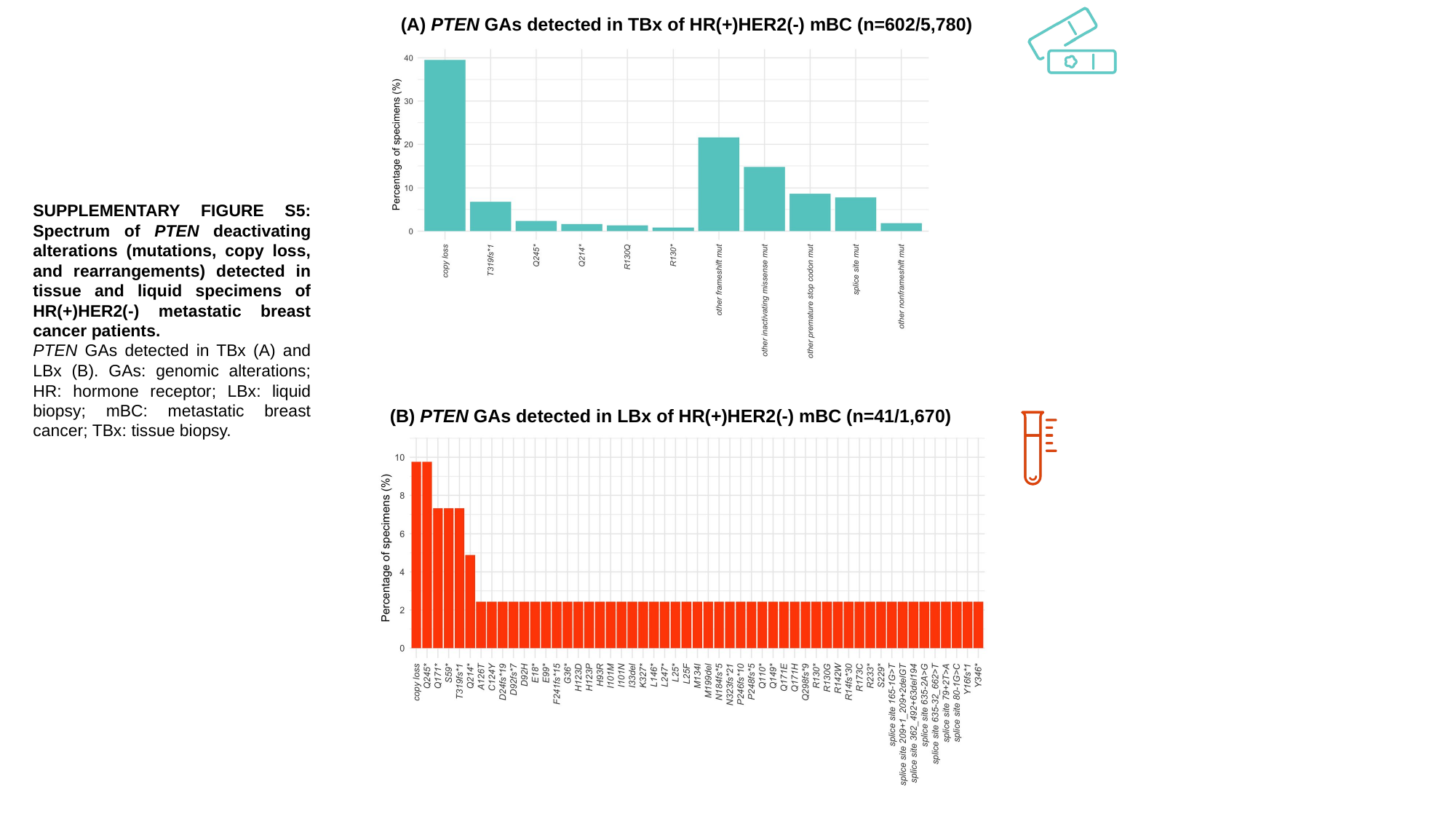

(A) PTEN GAs detected in TBx of HR(+)HER2(-) mBC (n=602/5,780)
SUPPLEMENTARY FIGURE S5: Spectrum of PTEN deactivating alterations (mutations, copy loss, and rearrangements) detected in tissue and liquid specimens of HR(+)HER2(-) metastatic breast cancer patients.
PTEN GAs detected in TBx (A) and LBx (B). GAs: genomic alterations; HR: hormone receptor; LBx: liquid biopsy; mBC: metastatic breast cancer; TBx: tissue biopsy.
(B) PTEN GAs detected in LBx of HR(+)HER2(-) mBC (n=41/1,670)

## Slide 7
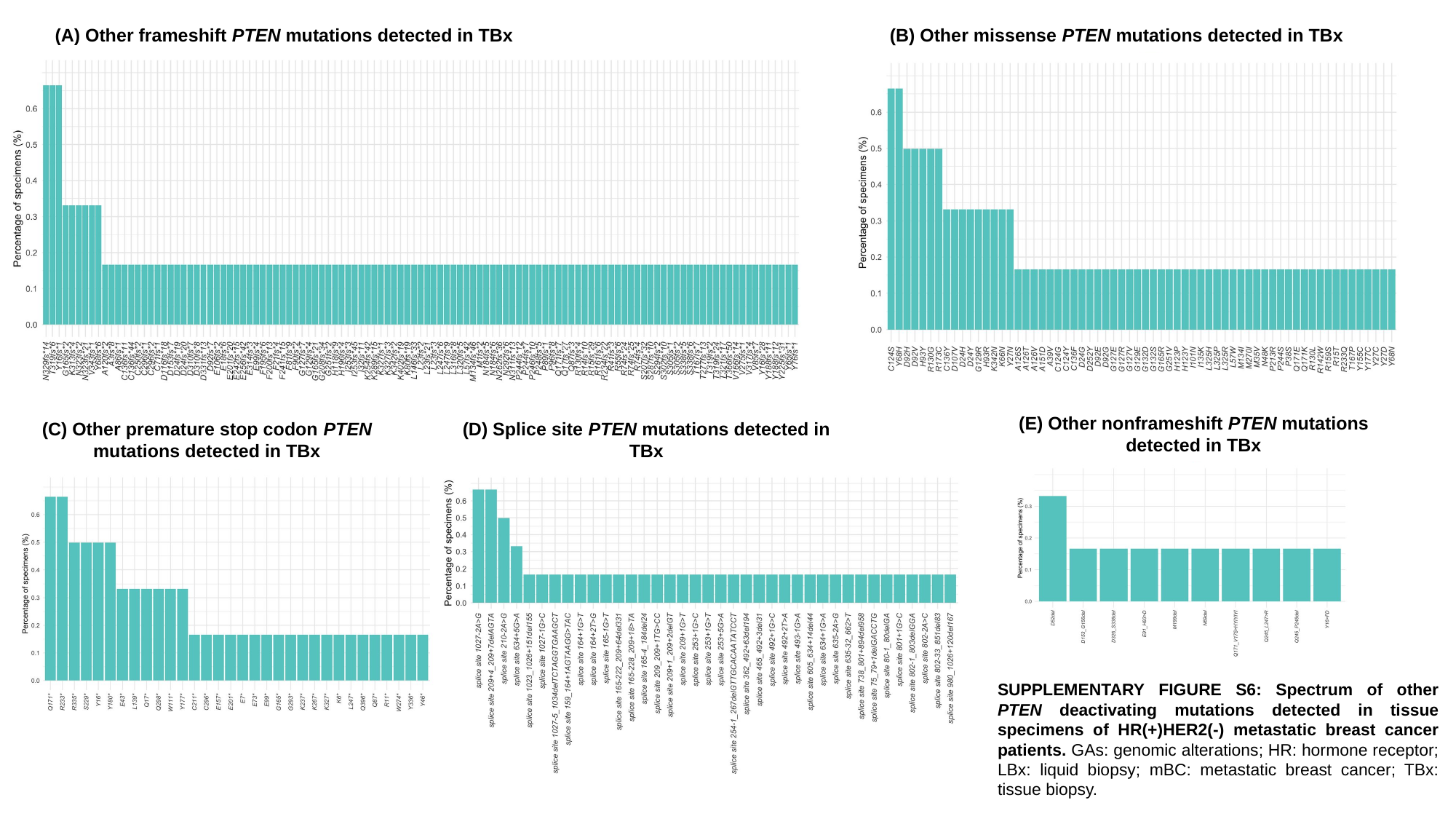

(B) Other missense PTEN mutations detected in TBx
(A) Other frameshift PTEN mutations detected in TBx
(E) Other nonframeshift PTEN mutations detected in TBx
(C) Other premature stop codon PTEN mutations detected in TBx
(D) Splice site PTEN mutations detected in TBx
SUPPLEMENTARY FIGURE S6: Spectrum of other PTEN deactivating mutations detected in tissue specimens of HR(+)HER2(-) metastatic breast cancer patients. GAs: genomic alterations; HR: hormone receptor; LBx: liquid biopsy; mBC: metastatic breast cancer; TBx: tissue biopsy.

## Slide 8
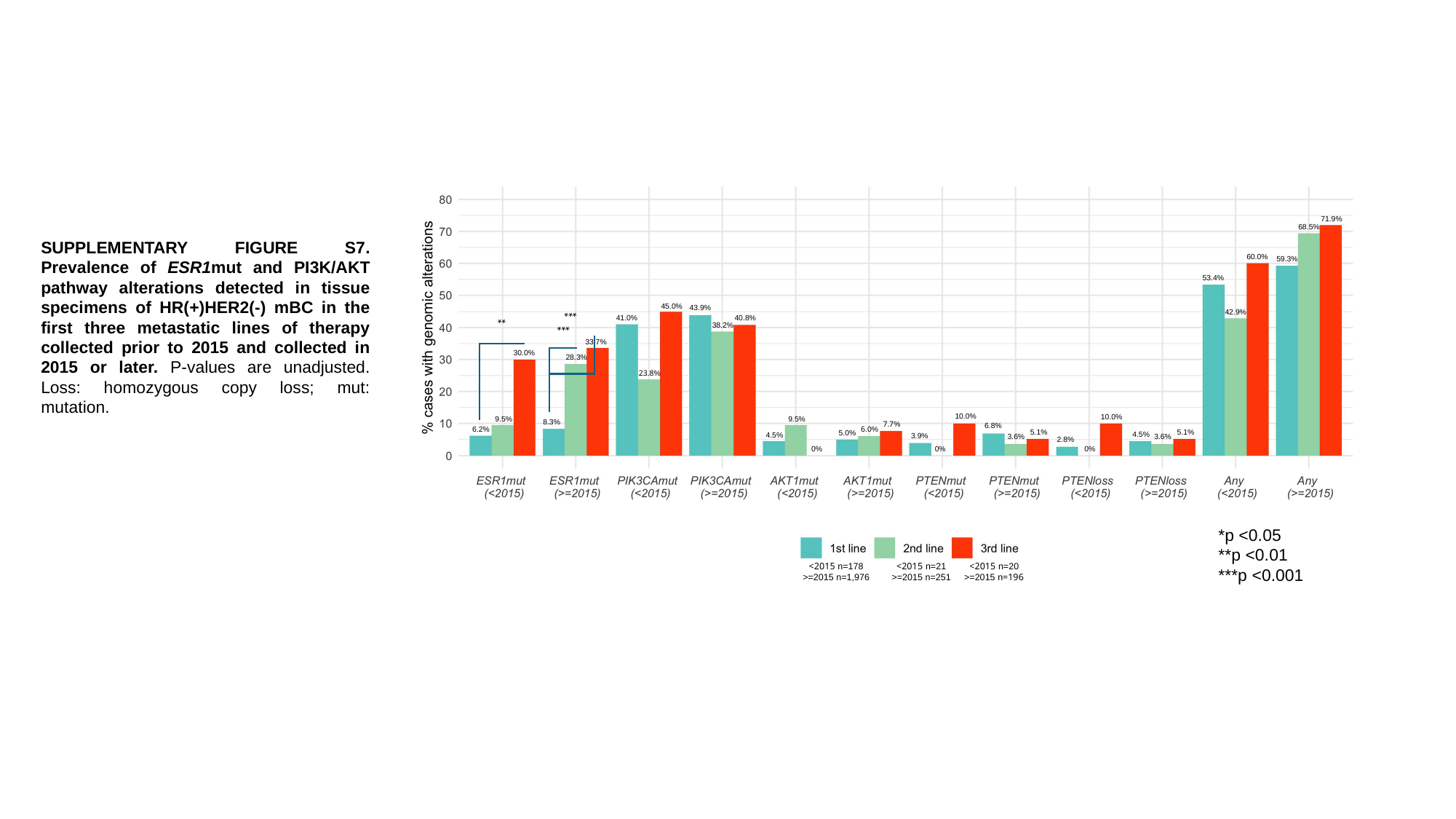

71.9%
68.5%
SUPPLEMENTARY FIGURE S7. Prevalence of ESR1mut and PI3K/AKT pathway alterations detected in tissue specimens of HR(+)HER2(-) mBC in the first three metastatic lines of therapy collected prior to 2015 and collected in 2015 or later. P-values are unadjusted. Loss: homozygous copy loss; mut: mutation.
60.0%
59.3%
53.4%
45.0%
43.9%
***
42.9%
41.0%
40.8%
**
38.2%
***
33.7%
30.0%
28.3%
23.8%
10.0%
10.0%
9.5%
9.5%
8.3%
7.7%
6.8%
6.0%
6.2%
5.1%
5.1%
5.0%
4.5%
4.5%
3.9%
3.6%
3.6%
2.8%
0%
0%
0%
*p <0.05
**p <0.01
***p <0.001
<2015 n=20
>=2015 n=196
<2015 n=21
>=2015 n=251
<2015 n=178
>=2015 n=1,976

## Slide 9
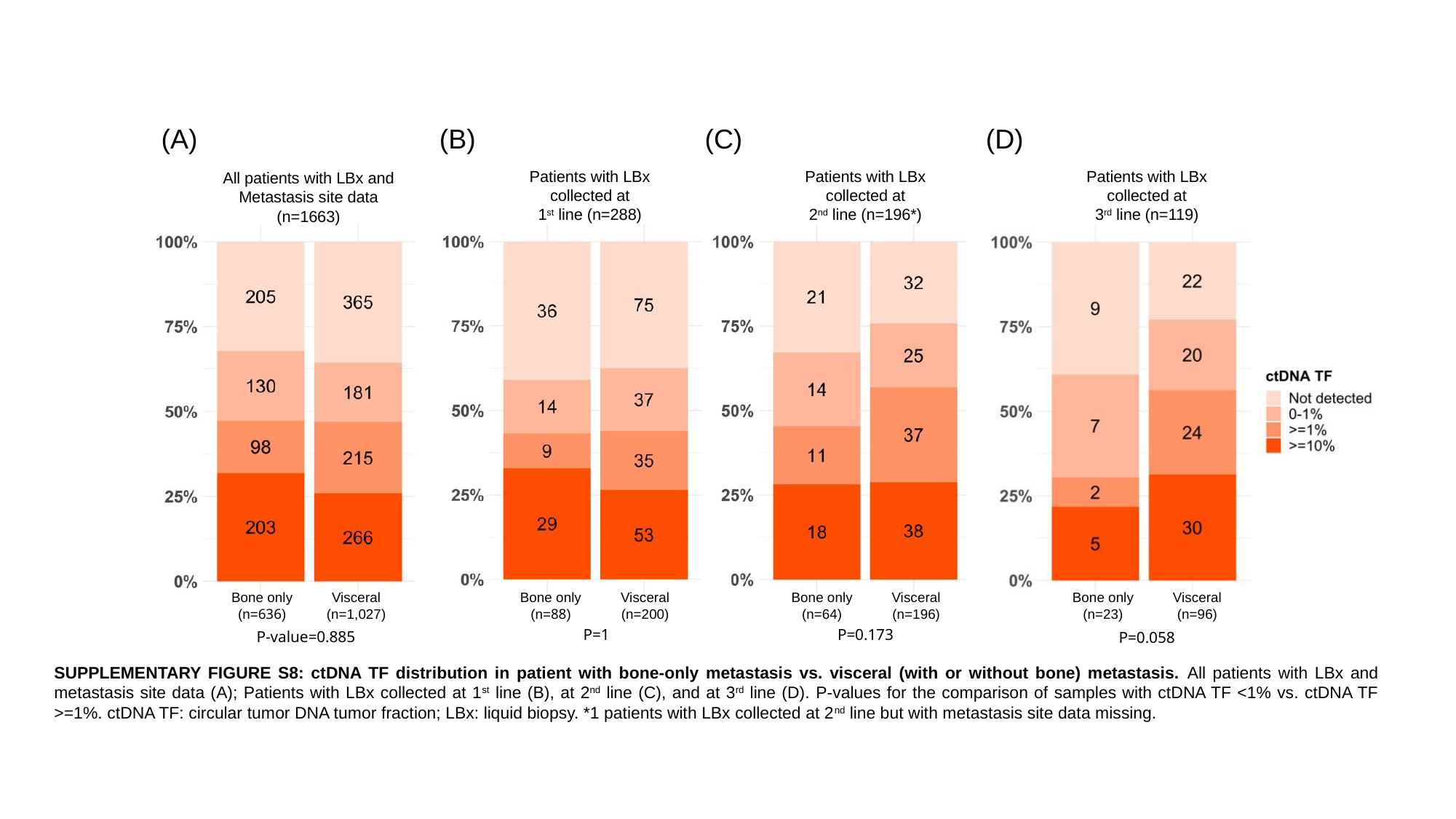

(A)
(B)
(C)
(D)
Patients with LBx collected at
2nd line (n=196*)
Patients with LBx collected at
1st line (n=288)
Patients with LBx collected at
3rd line (n=119)
All patients with LBx and Metastasis site data
(n=1663)
Visceral
(n=1,027)
Visceral
(n=200)
Visceral
(n=196)
Visceral
(n=96)
Bone only
(n=636)
Bone only
(n=88)
Bone only
(n=64)
Bone only
(n=23)
P=0.173
P=1
P-value=0.885
P=0.058
SUPPLEMENTARY FIGURE S8: ctDNA TF distribution in patient with bone-only metastasis vs. visceral (with or without bone) metastasis. All patients with LBx and metastasis site data (A); Patients with LBx collected at 1st line (B), at 2nd line (C), and at 3rd line (D). P-values for the comparison of samples with ctDNA TF <1% vs. ctDNA TF >=1%. ctDNA TF: circular tumor DNA tumor fraction; LBx: liquid biopsy. *1 patients with LBx collected at 2nd line but with metastasis site data missing.

## Slide 10
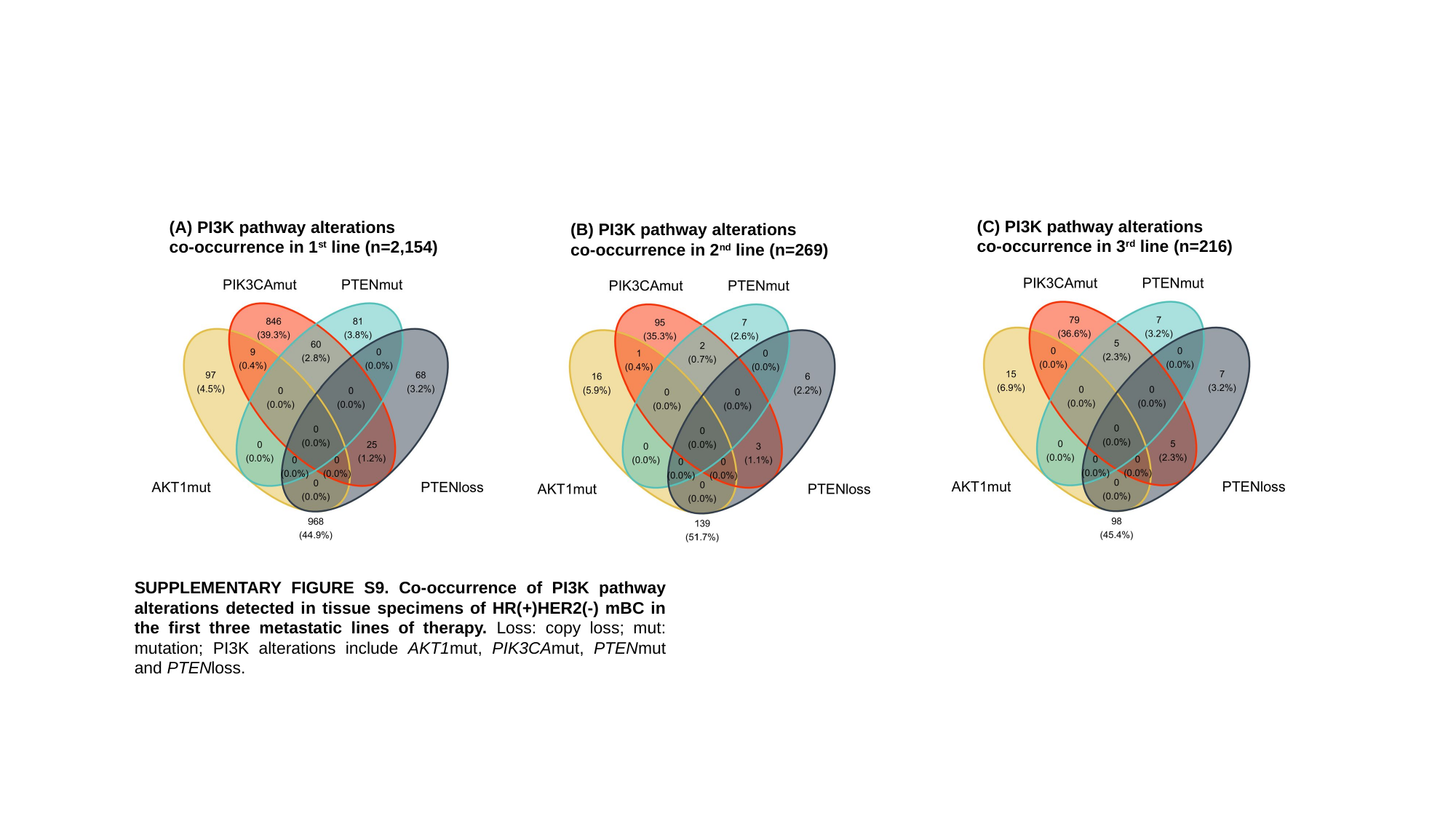

(C) PI3K pathway alterations
co-occurrence in 3rd line (n=216)
(A) PI3K pathway alterations
co-occurrence in 1st line (n=2,154)
(B) PI3K pathway alterations
co-occurrence in 2nd line (n=269)
SUPPLEMENTARY FIGURE S9. Co-occurrence of PI3K pathway alterations detected in tissue specimens of HR(+)HER2(-) mBC in the first three metastatic lines of therapy. Loss: copy loss; mut: mutation; PI3K alterations include AKT1mut, PIK3CAmut, PTENmut and PTENloss.

## Slide 11
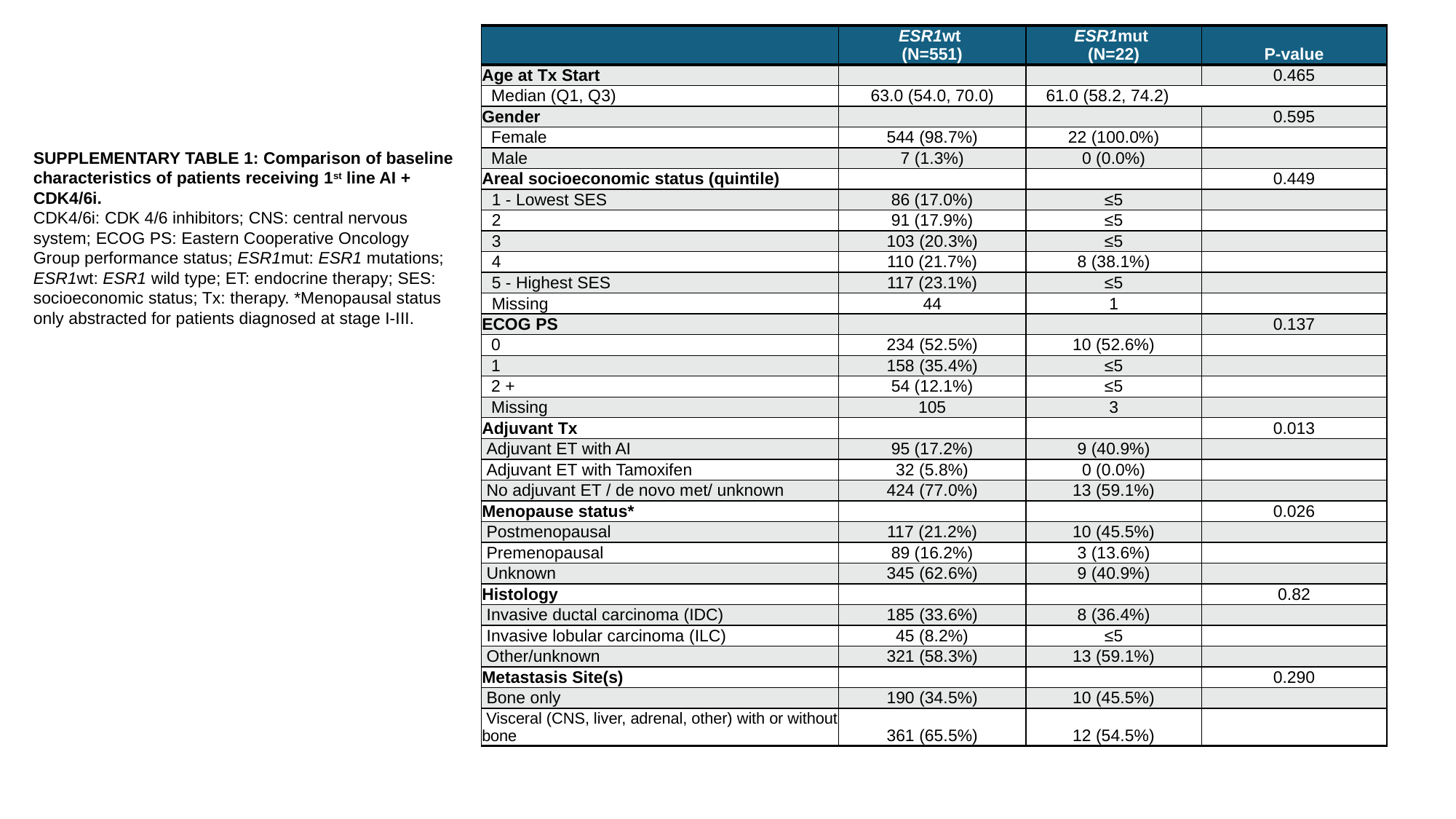

| | ESR1wt (N=551) | ESR1mut (N=22) | P-value |
| --- | --- | --- | --- |
| Age at Tx Start | | | 0.465 |
| Median (Q1, Q3) | 63.0 (54.0, 70.0) | 61.0 (58.2, 74.2) | |
| Gender | | | 0.595 |
| Female | 544 (98.7%) | 22 (100.0%) | |
| Male | 7 (1.3%) | 0 (0.0%) | |
| Areal socioeconomic status (quintile) | | | 0.449 |
| 1 - Lowest SES | 86 (17.0%) | ≤5 | |
| 2 | 91 (17.9%) | ≤5 | |
| 3 | 103 (20.3%) | ≤5 | |
| 4 | 110 (21.7%) | 8 (38.1%) | |
| 5 - Highest SES | 117 (23.1%) | ≤5 | |
| Missing | 44 | 1 | |
| ECOG PS | | | 0.137 |
| 0 | 234 (52.5%) | 10 (52.6%) | |
| 1 | 158 (35.4%) | ≤5 | |
| 2 + | 54 (12.1%) | ≤5 | |
| Missing | 105 | 3 | |
| Adjuvant Tx | | | 0.013 |
| Adjuvant ET with AI | 95 (17.2%) | 9 (40.9%) | |
| Adjuvant ET with Tamoxifen | 32 (5.8%) | 0 (0.0%) | |
| No adjuvant ET / de novo met/ unknown | 424 (77.0%) | 13 (59.1%) | |
| Menopause status\* | | | 0.026 |
| Postmenopausal | 117 (21.2%) | 10 (45.5%) | |
| Premenopausal | 89 (16.2%) | 3 (13.6%) | |
| Unknown | 345 (62.6%) | 9 (40.9%) | |
| Histology | | | 0.82 |
| Invasive ductal carcinoma (IDC) | 185 (33.6%) | 8 (36.4%) | |
| Invasive lobular carcinoma (ILC) | 45 (8.2%) | ≤5 | |
| Other/unknown | 321 (58.3%) | 13 (59.1%) | |
| Metastasis Site(s) | | | 0.290 |
| Bone only | 190 (34.5%) | 10 (45.5%) | |
| Visceral (CNS, liver, adrenal, other) with or without bone | 361 (65.5%) | 12 (54.5%) | |
SUPPLEMENTARY TABLE 1: Comparison of baseline characteristics of patients receiving 1st line AI + CDK4/6i.
CDK4/6i: CDK 4/6 inhibitors; CNS: central nervous system; ECOG PS: Eastern Cooperative Oncology Group performance status; ESR1mut: ESR1 mutations; ESR1wt: ESR1 wild type; ET: endocrine therapy; SES: socioeconomic status; Tx: therapy. *Menopausal status only abstracted for patients diagnosed at stage I-III.

## Slide 12
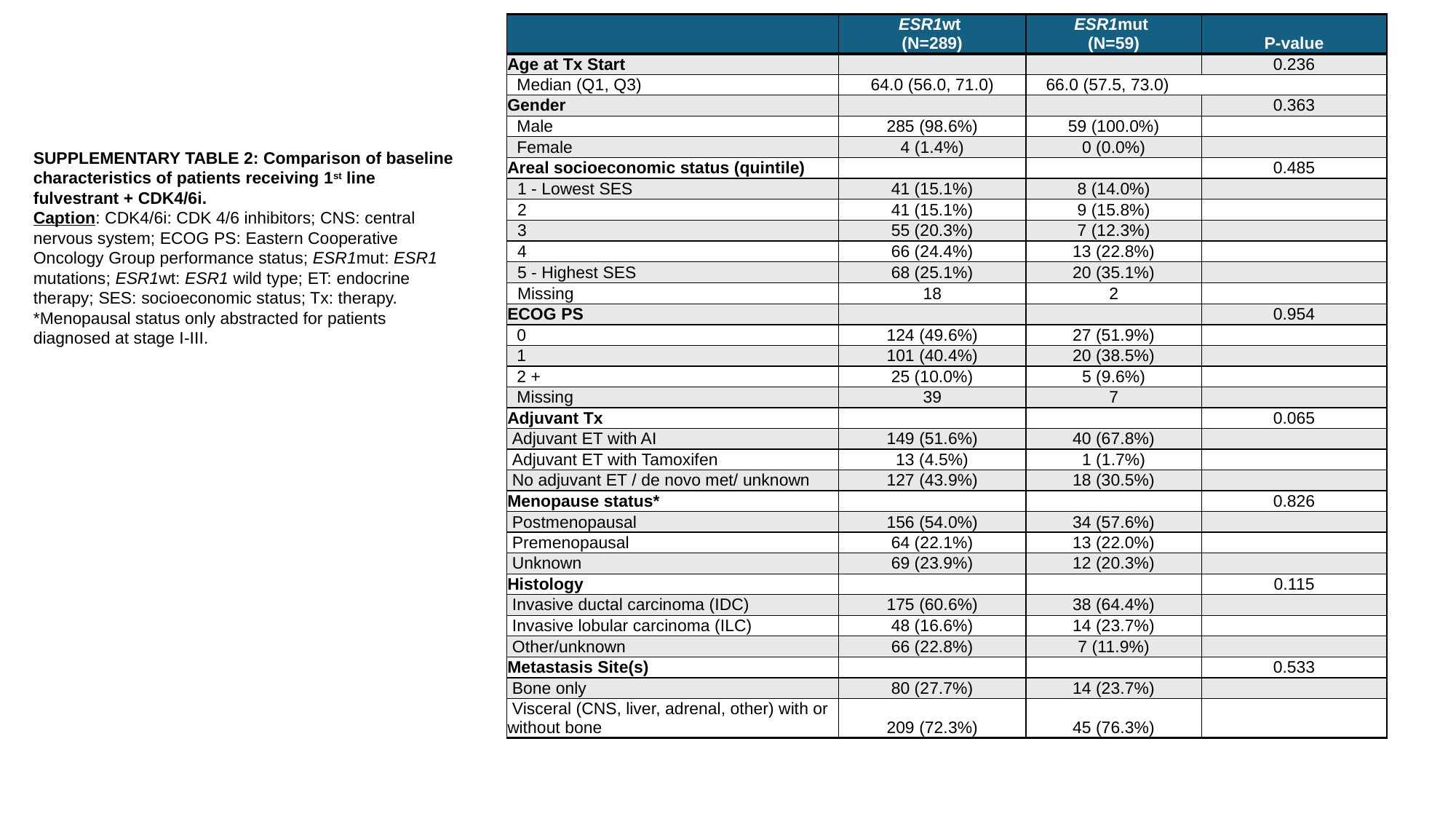

| | ESR1wt (N=289) | ESR1mut (N=59) | P-value |
| --- | --- | --- | --- |
| Age at Tx Start | | | 0.236 |
| Median (Q1, Q3) | 64.0 (56.0, 71.0) | 66.0 (57.5, 73.0) | |
| Gender | | | 0.363 |
| Male | 285 (98.6%) | 59 (100.0%) | |
| Female | 4 (1.4%) | 0 (0.0%) | |
| Areal socioeconomic status (quintile) | | | 0.485 |
| 1 - Lowest SES | 41 (15.1%) | 8 (14.0%) | |
| 2 | 41 (15.1%) | 9 (15.8%) | |
| 3 | 55 (20.3%) | 7 (12.3%) | |
| 4 | 66 (24.4%) | 13 (22.8%) | |
| 5 - Highest SES | 68 (25.1%) | 20 (35.1%) | |
| Missing | 18 | 2 | |
| ECOG PS | | | 0.954 |
| 0 | 124 (49.6%) | 27 (51.9%) | |
| 1 | 101 (40.4%) | 20 (38.5%) | |
| 2 + | 25 (10.0%) | 5 (9.6%) | |
| Missing | 39 | 7 | |
| Adjuvant Tx | | | 0.065 |
| Adjuvant ET with AI | 149 (51.6%) | 40 (67.8%) | |
| Adjuvant ET with Tamoxifen | 13 (4.5%) | 1 (1.7%) | |
| No adjuvant ET / de novo met/ unknown | 127 (43.9%) | 18 (30.5%) | |
| Menopause status\* | | | 0.826 |
| Postmenopausal | 156 (54.0%) | 34 (57.6%) | |
| Premenopausal | 64 (22.1%) | 13 (22.0%) | |
| Unknown | 69 (23.9%) | 12 (20.3%) | |
| Histology | | | 0.115 |
| Invasive ductal carcinoma (IDC) | 175 (60.6%) | 38 (64.4%) | |
| Invasive lobular carcinoma (ILC) | 48 (16.6%) | 14 (23.7%) | |
| Other/unknown | 66 (22.8%) | 7 (11.9%) | |
| Metastasis Site(s) | | | 0.533 |
| Bone only | 80 (27.7%) | 14 (23.7%) | |
| Visceral (CNS, liver, adrenal, other) with or without bone | 209 (72.3%) | 45 (76.3%) | |
SUPPLEMENTARY TABLE 2: Comparison of baseline characteristics of patients receiving 1st line fulvestrant + CDK4/6i.
Caption: CDK4/6i: CDK 4/6 inhibitors; CNS: central nervous system; ECOG PS: Eastern Cooperative Oncology Group performance status; ESR1mut: ESR1 mutations; ESR1wt: ESR1 wild type; ET: endocrine therapy; SES: socioeconomic status; Tx: therapy. *Menopausal status only abstracted for patients diagnosed at stage I-III.

## Slide 13
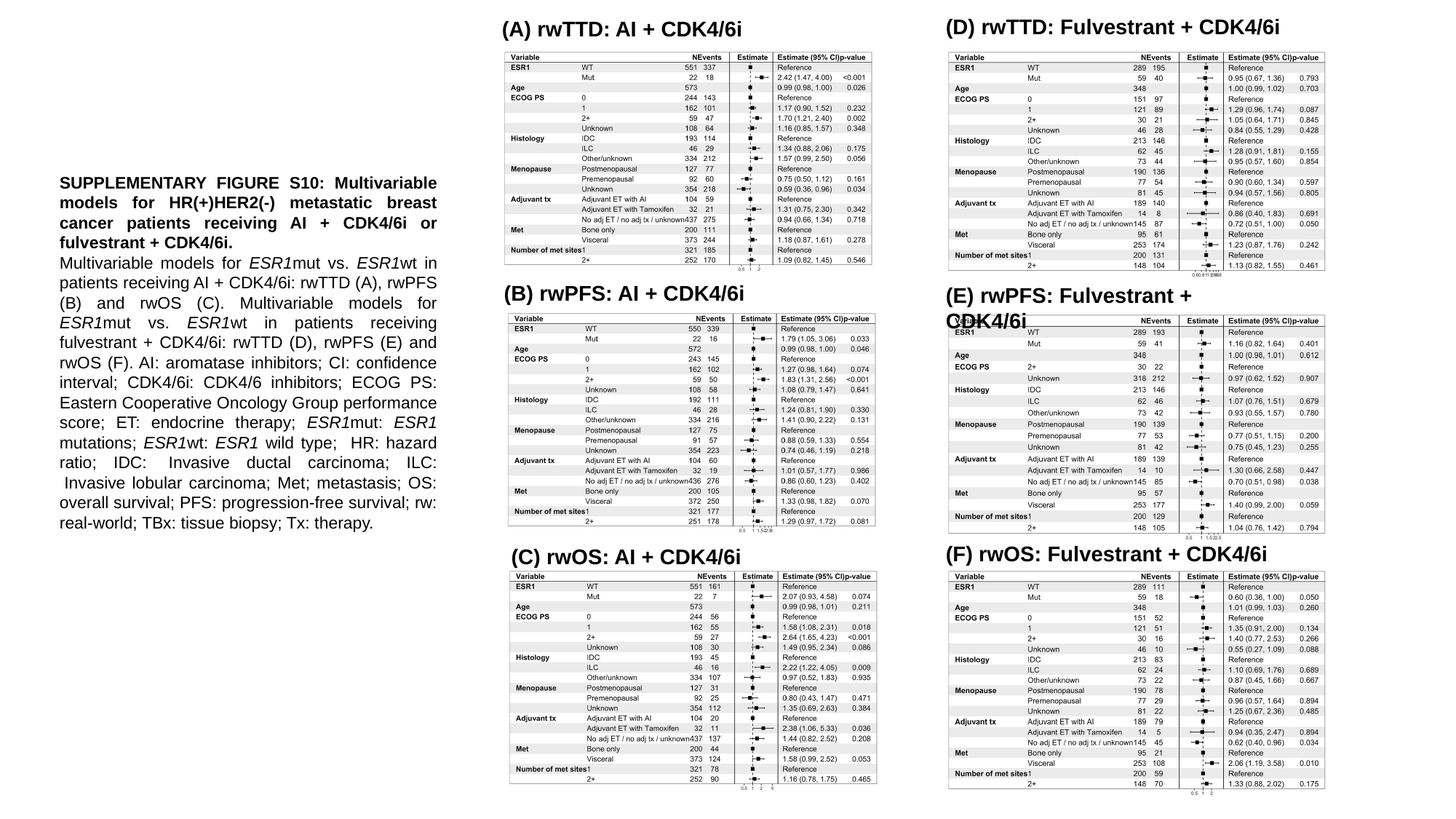

(D) rwTTD: Fulvestrant + CDK4/6i
(A) rwTTD: AI + CDK4/6i
SUPPLEMENTARY FIGURE S10: Multivariable models for HR(+)HER2(-) metastatic breast cancer patients receiving AI + CDK4/6i or fulvestrant + CDK4/6i.
Multivariable models for ESR1mut vs. ESR1wt in patients receiving AI + CDK4/6i: rwTTD (A), rwPFS (B) and rwOS (C). Multivariable models for ESR1mut vs. ESR1wt in patients receiving fulvestrant + CDK4/6i: rwTTD (D), rwPFS (E) and rwOS (F). AI: aromatase inhibitors; CI: confidence interval; CDK4/6i: CDK4/6 inhibitors; ECOG PS: Eastern Cooperative Oncology Group performance score; ET: endocrine therapy; ESR1mut: ESR1 mutations; ESR1wt: ESR1 wild type; HR: hazard ratio; IDC:  Invasive ductal carcinoma; ILC:  Invasive lobular carcinoma; Met; metastasis; OS: overall survival; PFS: progression-free survival; rw: real-world; TBx: tissue biopsy; Tx: therapy.
(B) rwPFS: AI + CDK4/6i
(E) rwPFS: Fulvestrant + CDK4/6i
(F) rwOS: Fulvestrant + CDK4/6i
(C) rwOS: AI + CDK4/6i
